# A Novel Longitudinal Proteomic Aging Index Predicts Mortality, Multimorbidity, and Frailty in Older Adults

**DOI:** 10.1111/acel.70317

**Published:** 2025-12-08

**Authors:** Zexi Rao, Shuo Wang, Aixin Li, Michael J. Blaha, Josef Coresh, Peter Ganz, Catherine H. Marshall, James S. Pankow, Elizabeth A. Platz, Wendy Post, Sanaz Sedaghat, Jerome I. Rotter, Seamus P. Whelton, Anna Prizment, Weihua Guan

**Affiliations:** ^1^ Division of Biostatistics and Health Data Science University of Minnesota Minneapolis Minnesota USA; ^2^ Department of Laboratory Medicine and Pathology University of Minnesota Minneapolis Minnesota USA; ^3^ Division of Epidemiology and Community Health University of Minnesota Minneapolis Minnesota USA; ^4^ Johns Hopkins Ciccarone Center for Prevention of Cardiovascular Disease Johns Hopkins University School of Medicine Baltimore Maryland USA; ^5^ Departments of Population Health and Medicine NYU Grossman School of Medicine New York New York USA; ^6^ Division of Cardiology, Zuckerberg San Francisco General Hospital and Department of Medicine University of California San Francisco California USA; ^7^ Sidney Kimmel Comprehensive Cancer Center at Johns Hopkins Baltimore Maryland USA; ^8^ Department of Epidemiology Johns Hopkins Bloomberg School of Public Health Baltimore Maryland USA; ^9^ Institute for Translational Genomics and Population Sciences, The Lundquist Institute for Biomedical Innovation, Department of Pediatrics Harbor‐UCLA Medical Center Torrance California USA

**Keywords:** biological aging, biomarkers, frailty, longitudinal studies, mortality, multimorbidity, proteomics

## Abstract

Previous studies have developed proteomic aging clocks to estimate biological age and predict mortality and age‐related diseases. However, these earlier clocks were based on cross‐sectional data, capturing only the cumulative aging burden at a single time point but were unable to reflect the dynamic trajectory of biological aging over time. We constructed a longitudinal proteomic aging index (LPAI) using data from 4684 plasma proteins measured by the SomaScan 5K Array across three visits in the Atherosclerosis Risk in Communities (ARIC) study (ages 67–90 at last visit). Our two‐step approach applied functional principal component analysis (FPCA) to capture protein‐level change patterns over time, followed by elastic net penalized Cox regression for protein selection. LPAI was constructed in a randomly selected training set of ARIC participants (*N* = 2954), tested among the remaining ARIC participants (*N* = 1267), and validated externally in Multi‐Ethnic Study of Atherosclerosis (MESA) participants (*N* = 3726, ages 53–94 at last exam). Using Cox proportional hazards model, higher LPAI was associated with increased all‐cause mortality (HR = 2.50, 95% CI: [2.15, 2.92] per SD), CVD mortality (HR = 1.79, 95% CI: [1.34, 2.39] per SD), and cancer mortality (HR = 1.96, 95% CI: [1.45, 2.64] per SD) risk in ARIC, with statistically significant and directionally consistent associations also observed in MESA. Additionally, higher LPAI was associated with increased multimorbidity and frailty. This study demonstrates the feasibility of developing biological aging measures from longitudinal proteomics data and supports LPAI as a biomarker for aging‐related health risks.

## Graphical abstract

We used proteomic data from three longitudinal visits in the ARIC study to construct a single longitudinal proteomic aging index (LPAI) that captures both cumulative aging burden and dynamic aging trajectories. Higher LPAI was associated with increased risks of mortality, frailty, and multimorbidity.

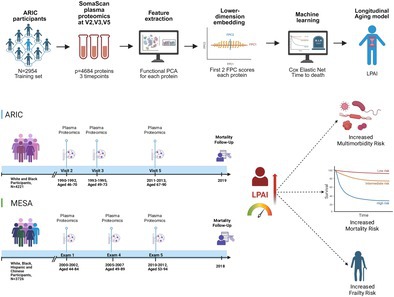

## 1 Introduction

Deterioration in physiological functioning with age is a significant contributor to increased mortality rates in older populations, as declining organ function can lead to life‐threatening conditions and reduced resilience to health challenges (Young 1997).

Biological aging research is essential for understanding the natural processes that cause the decline in physiological functioning and heightened vulnerability to disease. For example, one prominent manifestation of this decline is multimorbidity, defined as the coexistence of two or more chronic disease conditions, which is prevalent among older adults and becomes increasingly common with advancing age (Navickas et al. 2016). Increased multimorbidity reduces the quality of life and is associated with elevated mortality risk. Another consequence is frailty, a clinical syndrome that includes weight loss, exhaustion, weakness, slow walking speed, and low physical activity (Fried et al. 2001), which raises the risk of adverse outcomes, including falls, hospitalization, and death (Xue 2011).

Previously, several biological aging measures based on omics data have been developed, with the epigenetic clock being the most extensively studied (Hannum et al. 2013; Horvath 2013; Levine et al. 2015; Lu et al. 2019). Epigenetic aging clocks, derived from DNA methylation biomarkers in blood or tissue, have shown consistent associations with mortality risk (Lu et al. 2019), and have also been linked to increased risk of overall cancer (Levine et al. 2015, 2018), and multimorbidity (Jain et al. 2023). More recently, proteomic aging clocks (PAC), which combine a set of proteomic‐based aging‐related biomarkers, have emerged as promising biological age estimators (Lehallier et al. 2020; Tanaka et al. 2018). Because proteins offer direct insights into biological processes associated with many physiological and pathological aspects of aging (Zaghlool et al. 2020), and respond more dynamically to environmental influences than epigenetic markers, they are well‐suited as biomarkers for monitoring aging trajectories (Kliuchnikova et al. 2024). This underscores the promise of proteomic‐based aging measures as alternatives to epigenetic clocks and traditional clinical biomarker–based age estimates, such as BioAge, derived using the Klemera–Doubal method (KDM) (Klemera and Doubal 2006), and PhenoAge (Liu et al. 2019), which integrates predefined clinical biomarkers. Recent evidence further suggests that protein‐based aging clocks capture aspects of aging biology that are not well represented by epigenetic aging clocks (Argentieri et al. 2024).

**FIGURE 1:**
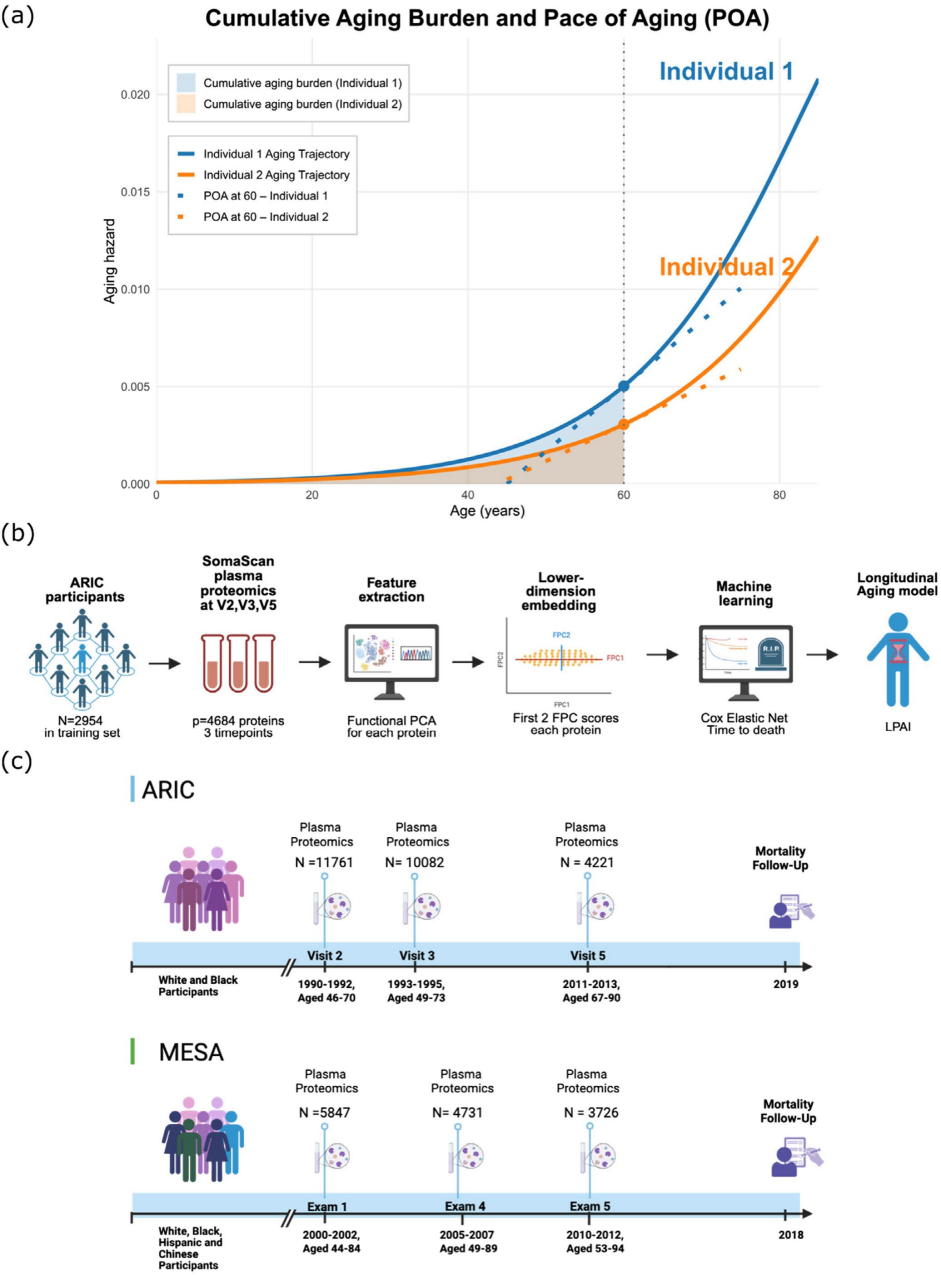
Study design and conceptual framework for Longitudinal Proteomic Aging Index (LPAI) construction. (a) Conceptual illustration of the relationship between aging hazard (solid curves), cumulative aging burden (shaded area), and the pace of aging (dotted tangent lines) for two individuals. The *x*‐axis represents chronological age, while the *y*‐axis represents aging hazard, indicating the instantaneous biological deterioration occurring at each age. The shaded areas under the curves quantify each individual's cumulative biological burden accrued up to age 60, while the dotted tangent lines show the slope of the aging trajectory, reflecting the pace of aging at age 60. Individual 1 exhibits both a higher cumulative aging burden and pace of aging at age 60. Together, these components illustrate how cumulative burden and pace of aging capture distinct yet complementary dimensions of the biological aging process. (b) Workflow for LPAI training. 2954 ARIC participants with proteomic data at all three visits were used as the training set. Longitudinal expression data for each protein was modeled using functional principal component analysis (FPCA) to extract major aging‐related components (FPC1, FPC2). The resulting first 2 FPC scores were input into a Cox elastic net regression to identify components associated with all‐cause mortality by shrinking the coefficients of non‐informative FPCs to zero. LPAI was defined as the log hazard ratio predicted by the fitted Cox model. (c) Follow‐up timeline of the ARIC and MESA studies. In ARIC, plasma samples were collected at Visit 2 (1990–1992), Visit 3 (1993–1995), and Visit 5 (2011–2013), with mortality follow‐up in 2019. A total of 4221 participants had proteomic measurements available at all three visits. In MESA, proteomics was conducted using samples from Exam 1 (2000–2002), Exam 4 (2005–2007), and Exam 5 (2010–2012), with mortality follow‐up in 2018. A total of 3726 participants had proteomic data from all three exams. Created in https://BioRender.com.

Aging is a dynamic and continuous process. Protein levels change with age and exhibit longitudinal within‐person variability in their trajectories (Dodig‐Crnković et al. 2020). Ideally, biological aging measurements should both measure the extent of damage accumulated with aging in an individual and, when assessed longitudinally, track the pace or trajectory at which this damage accrues over a certain period (Levine 2013). Recent PACs have been developed in large cohorts, including the Atherosclerosis Risk in Communities (ARIC) study and the UK Biobank, and have shown strong associations with mortality (Kuo et al. 2024; Wang et al. 2024), frailty, and other common age‐related diseases (Argentieri et al. 2024; Carrasco‐Zanini et al. 2024; Gadd et al. 2024). However, these existing PACs all rely on proteomic data measured at a single time point and are trained on cross‐sectional association with age or mortality. Therefore, they primarily capture the cumulative biological aging burden up to that measurement but fail to capture the dynamic rate and trajectory by which aging progresses over time. As illustrated conceptually in Figure 1a, the cumulative burden reflects the total biological damage accumulated with age, whereas another dimension of aging captures the rate at which biological damage occurs.

To quantify the latter, the Pace of Aging (POA) framework was proposed, integrating multiple longitudinal biomarker measurements to distinguish faster and slower agers within a cohort (Belsky et al. 2015). Using a mixed‐effects model, it derives an individual‐specific rate of physiological decline that serves as a quantitative indicator of biological aging and shows association with physical and cognitive functioning. This underscores the added value of incorporating longitudinal biomarker data in biological aging measures. However, because POA focuses solely on the rate of change in biomarkers, it does not capture the cumulative biological burden that has accumulated up to the time of measurement. Therefore, neither cross‐sectional clocks nor POA measures can simultaneously capture both cumulative burden and longitudinal trajectory of aging. Moreover, both POA and PAC are based on linear models. Prior studies have showed that there might exist non‐linear dynamics in aging phenotypes and non‐linear shifts in gene expression (Oh et al. 2023; Schaum et al. 2020) and age‐related inflection points in proteomic trajectories at ages 34, 60, and 78 years (Lehallier et al. 2019).

These limitations highlight the need for a more flexible, data‐driven approach to measure biological aging that jointly considers accumulated damage, dynamic trajectories, and potential non‐linear patterns of change. Repeated protein measurements provide a unique opportunity to study temporal patterns of proteomic aging, revealing aging mechanisms missed in cross‐sectional analyses. The primary objective of this study was to develop a novel longitudinal proteomic aging index (LPAI). We employed a two‐step approach framework: first, functional principal component analysis (FPCA) (Jones and Rice 1992) was used to capture both accumulated proteomic changes with aging and the trajectories underlying these changes over time; second, elastic net penalized Cox proportional hazards regression (Simon et al. 2011) was applied to select proteins most significantly associated with aging and mortality risk. This integrated framework identified individuals with distinct temporal protein expression patterns, characterized either by excessive accumulation of proteomic changes or by fluctuations in expression levels within specific age ranges. As a result, LPAI provides complementary insights not covered by existing PACs or POA measures.

We applied this approach to data from the ARIC study, which measured blood protein levels at three study visits using the SomaScan 5K Array (Wright et al. 2021). ARIC participants who had protein measurements at all three visits were included (*N* = 4221, age range 67–90 at last visit). To assess generalizability, we validated the LPAI in an external cohort: the Multi‐Ethnic Study of Atherosclerosis (MESA) (MESA 2019). MESA participants also had three protein measurements (*N* = 3726, age range 53–94 at last exam). We then evaluated the associations of LPAI with all‐cause, cancer, and cardiovascular (CVD) mortality in both cohorts, as well as with multimorbidity and frailty in ARIC, to assess its relevance as a biological indicator of aging‐related health outcomes.

## 2 Results

### 2.1 Clustering Age‐Related Protein Level Changes Using LOESS Reveals Non‐Linear Trends and Enriched Pathways

**FIGURE 2:**
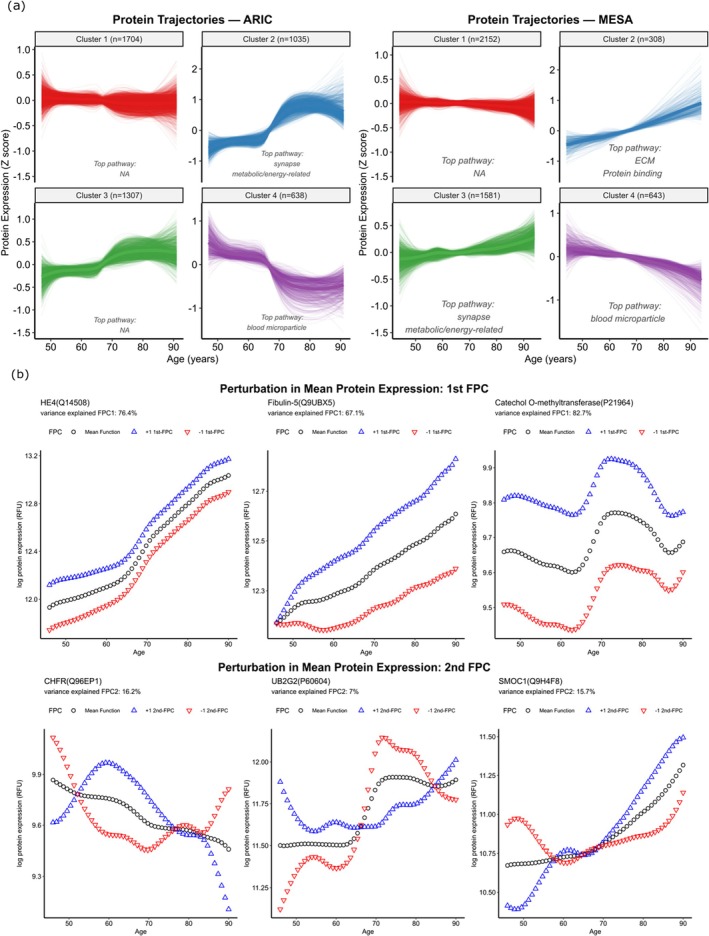
Age‐related protein expression trajectories and perturbation curves of mean protein expression level using functional principal components. (a) Four clusters of protein aging trajectories in ARIC and MESA. *Z*‐scored protein expression trajectories across age are shown for ARIC (left) and MESA (right). Each curve represents a protein's LOESS‐fitted trajectory, grouped into four clusters by K‐means clustering. Bold lines indicate the mean trajectory per cluster. Top GO‐enriched pathways for each cluster are shown; detailed protein and pathway list for each cluster are provided in the Supporting Information. (b) Perturbation of mean protein expression of FPCs in ARIC training set. Shown are proteins with non‐zero coefficients in the LPAI model, including the three with the largest weights on FPC1 and FPC2. The mean function (black) and ±1st/2nd FPC perturbations (blue/red) are displayed. The variance explained by each FPC is indicated above each panel. Mean function (black), ±1st FPC perturbation, and ±2nd FPC perturbation (+ blue −red), are shown. FPC, functional principal component.

To explore different patterns in proteomic aging, we clustered protein trajectories derived from LOESS‐fitted curves using k‐means. In both ARIC and MESA, four distinct clusters of age‐related protein expression patterns were identified (Figure 2a). The largest cluster (cluster 1) showed little change in protein levels across age in both cohorts. In contrast, proteins in clusters 2, 3, and 4 exhibited changes across the lifespan, with LOESS‐fitted trajectories showing the most pronounced non‐linear shifts around age 65 based on visual examination of the fitted curves.

We also conducted pathway enrichment analyses using Gene Ontology (GO) databases for each cluster. In ARIC, clusters 2 and 4 showed significant enrichment for biological pathways. Cluster 2 was enriched for synapse‐related processes and metabolic/energy‐related pathways, whereas top pathways in cluster 4 were predominantly enriched for blood microparticle. In MESA, cluster 3 showed enrichment for synapse‐related processes and metabolic/energy‐related pathways, mirroring the patterns observed in ARIC cluster 2, while cluster 4 again showed strong enrichment for blood microparticle pathways, consistent with ARIC cluster 4. Additionally, cluster 2 in MESA was enriched for extracellular matrix–related and protein‐binding pathways (e.g., glycosaminoglycan and heparin binding). Detailed lists of proteins within each cluster and their associated pathways are provided in the Supporting Information.

### 2.2 LPAI Construction and Validation Using FPCA and Elastic Net Cox Regression

We randomly divided the ARIC participants into training and test sets with a 70–30 split to construct and validate the LPAI. The training set of ARIC was composed of 2954 participants, of which 83.8% were White and 16.2% were Black. The mean age of the participants at Visit 5 was 76.2 years old (age range 67–90), and 57.5% were female. The test set of ARIC was composed of 1267 participants, of which 83.2% were White and 16.8% were Black. The mean age of the participants at Visit 5 was 76.1 years old (age range 67–90), and 59.0% were female. In the MESA validation set, 3726 participants were included that had proteomic data at all three exams. The mean age of MESA participants in the validation set at Exam 5 was 70.1 years old (age range 53–94), and 47.6% were female.

**FIGURE 3:**
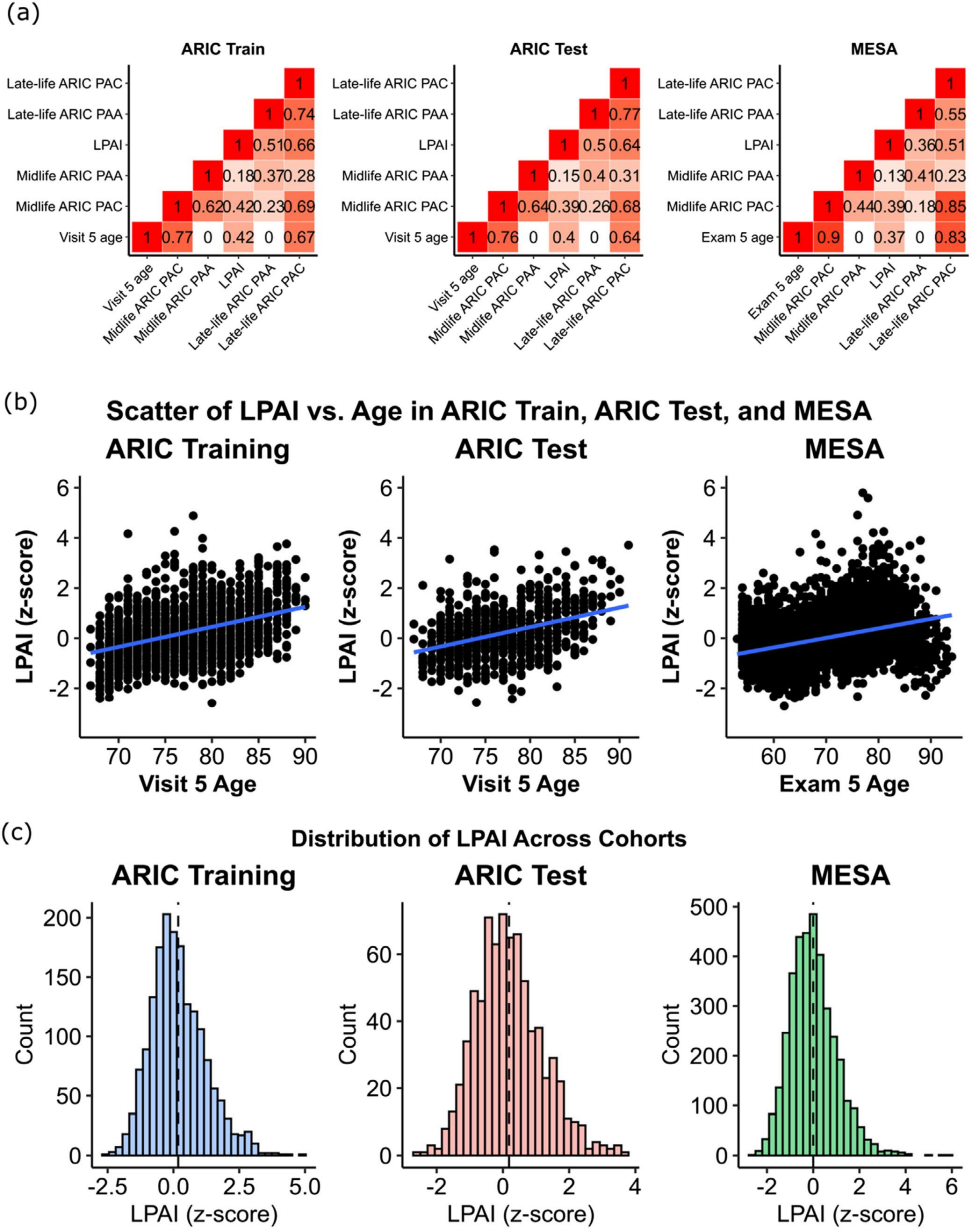
Association between LPAI and chronological age, and its correlation with other proteomic aging measures. (a) Pearson correlation between LPAI, chronological age (Visit 5 or Exam 5), and previously defined proteomic aging measures (PAAs and PACs) across ARIC training, ARIC test, and MESA. (b) Scatter plots showing the association between LPAI (*z*‐scored) and age at Visit 5 or Exam 5 across ARIC training, ARIC test, and MESA. (c) Histograms of LPAI in ARIC training, ARIC test, and MESA cohorts demonstrate approximately normal distributions. PAC, proteomic age clock; PAA, proteomic age acceleration.

We applied FPCA to extract functional principal component (FPC) scores for each of the 4684 proteins in the ARIC training set. Across all proteins, the first two FPCs explained an average of 90.3% of the cumulative variance. The first two FPC scores were then used as input features in an Elastic Net Penalized Cox Proportional Hazards Regression model, with time‐to‐death from all‐cause mortality as the outcome. After training, 185 FPCs corresponding to 181 unique proteins were assigned non‐zero coefficients. The full list of these 181 proteins is provided in the Supporting Information. LPAI was calculated as the weighted sum of the FPC scores, with weights determined by the Cox model coefficients. LPAI was approximately normally distributed and standardized (*z*‐scored) for analysis (Figure 3c). For visualization and descriptive purposes, LPAI was also dichotomized at the cohort‐specific median into high and low groups. Table 1 summarizes baseline characteristics of participants in ARIC and MESA at Visit/Exam 5 by LPAI group. In both cohorts, LPAI values showed a modest positive correlation with chronological age, indicating that individuals with higher LPAI tended to be older (Figure 3a,b).

**TABLE 1:** Characteristics of ARIC and MESA participants by median‐dichotomized LPAI (low vs. high) at visit/exam 5.

	ARIC test set			ARIC training set			MESA		
	Low LPAI (*N* = 633) −2.60 to −0.12	High LPAI (*N* = 634) −0.12 to 4.88	*p* value^c^	Low LPAI (*N* = 1477) −2.75 to −0.09	High LPAI (*N* = 1477) −0.09 to 3.71	*p* value^c^	Low LPAI (*N* = 1863) −2.72 to −0.09	High LPAI (*N* = 1863) −0.09 to 5.80	*p* value^c^
Mean age at visit/exam 5 (SD)	74.5 (4.3)	77.8 (5.2)	< 0.0001	74.5 (4.5)	77.8 (5.3)	< 0.0001	66.7 (8.9)	72.7 (8.9)	< 0.0001
Race									
White, %	81.4	85.0	0.096	84.0	83.5	0.76	38.4	44.6	< 0.0001
Black, %	18.6	5.0		16.0	16.5		25.2	25.6	
Chinese, %	NA	NA		NA	NA		14.8	7.6	
Hispanic, %	NA	NA		NA	NA		21.5	22.3	
Female, %	66.5	51.6	< 0.0001	65.5	49.5	< 0.0001	60.2	45.3	< 0.0001
Education level									
Less than high school graduate, %	9.4	14.8	< 0.0001	9.2	16.3	< 0.0001	12.0	16.3	< 0.0001
High school equivalent, %	40.1	49.1		41.6	44.3		16.5	19.1	
At least some college, %	50.6	36.1		49.2	39.4		72.2	64.6	
Smoking status									
Current smoker, %	3.2	7.8	0.0002	3.4	9.2	< 0.0001	3.8	12.2	< 0.0001
Former smoker, %	50.2	54.2		47.5	55.3		42.4	53.3	
Never smoker, %	46.6	40.0		49.1	35.6		53.9	34.5	
Alcohol consumption status^a^									
Current drinker, %	58.9	47.9	0.0002	54.2	47.7	< 0.0001	45.3	43.9	0.43
Former drinker, %	21.1	29.8		24.9	34.3		NA	NA	
Never drinker, %	20.0	22.3		20.9	17.9				
Diabetes, %	27.1	40.9	< 0.0001	23.1	39.4	< 0.0001	33.1	49.62	< 0.0001
Physical activity (SD)^b^	2.7 (0.8)	2.4 (0.8)	< 0.0001	2.7 (0.8)	2.5 (0.8)	< 0.0001	5471.2 (5821.0)	4845.7 (6188.3)	0.002
Hypertension, %	69.1	78.0	0.0004	66.3	79.2	< 0.0001	50.1	69.0	< 0.0001
Mean BMI, kg/m^2^ (SD)	28.3 (4.9)	28.9 (5.3)	0.068	28.3 (5.3)	28.9 (6.0)	0.004	28.2 (5.5)	28.9 (5.7)	< 0.0001
Mean eGFR, mL/min/1.73 m^2^ (SD)	76.8 (13.9)	67.0 (17.6)	< 0.0001	76.8 (14.1)	67.0 (18.8)	< 0.0001	69.7 (12.9)	64.3 (16.3)	< 0.0001

Abbreviations: BMI, body mass index; eGFR, estimated glomerular filtration rate; SD, standard deviation.

^a^ In MESA, alcohol consumption was classified as current drinker versus non‐drinker.

^b^ ARIC: Physical activity was assessed as a sport index during leisure time, ranged from 1 to 5. MESA: Physical activity defined by Weekly MET‐minutes, Unit: MET‐MIN/WK M‐SU.

^c^ Pearson's chi‐squared test; Welch two‐sample *t*‐test.

### 2.3 FPCs Captured Cumulative and Dynamic Components of Proteomic Aging

To illustrate the temporal aging patterns captured by the LPAI, we plotted the mean trajectories of selected proteins along with perturbed curves obtained by adding or subtracting one unit of the first or second functional principal component (FPC) function (Figure 2b). For illustration, we selected the three proteins with the largest weights on the first principal component (FPC1) and the three proteins with the largest weights on the second principal component (FPC2) in the elastic net Cox regression model. The black curve represents the mean trajectory, while the blue and red curves show the resulting deviations when the corresponding FPC functions are added or subtracted, respectively.

The first FPC primarily reflected overall shifts in protein levels, with perturbation curves that remained approximately parallel to the mean function, indicating coherent increases or decreases in expression across the lifespan. A higher positive FPC1 score indicated consistently higher‐than‐average protein expression across the lifespan. In contrast, the second FPC captured localized deviations from the mean, with effects more pronounced within specific age intervals, such as midlife or late life, corresponding to dynamic changes in the pace of aging that vary across time.

To assess cohort consistency, we also performed FPCA separately in ARIC and MESA for proteins with the largest FPC1 and FPC2 weights identified in LPAI. The estimated mean trajectories were generally similar but showed cohort‐specific differences for several proteins (Figure S5).

### 2.4 Pathway Enrichment and Mortality Associations of LPAI Proteins

**FIGURE 4:**
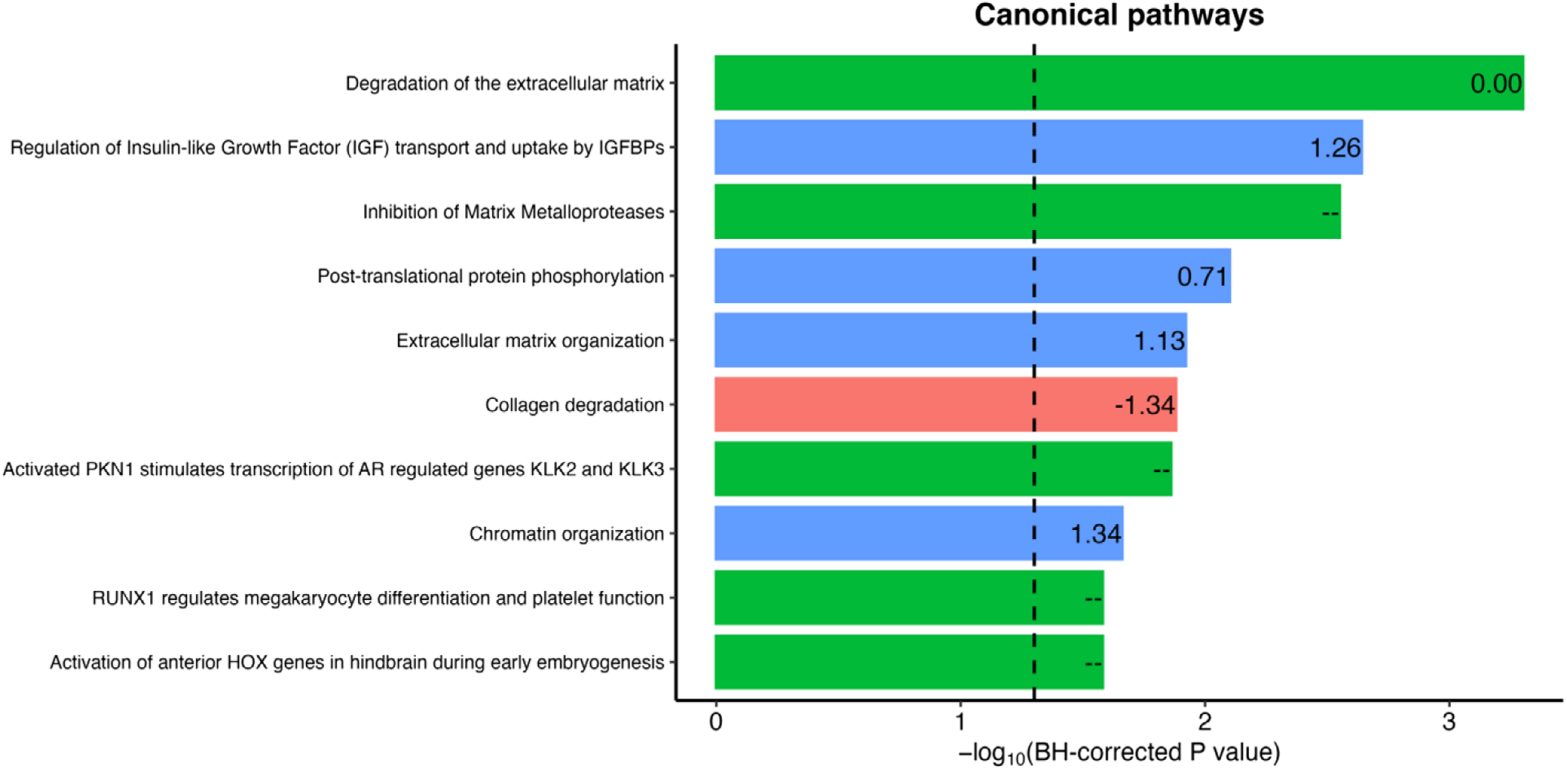
Top 10 significantly enriched pathways using ingenuity pathway analysis (IPA) with non‐zero proteins contributing to the LPAI. The background reference set included all 4684 proteins measured in ARIC. The figure presents the top 10 most significantly enriched pathways (FDR‐corrected *p* < 0.05). The numbers at the end of the bars represent the activation *z*‐scores calculated by the IPA algorithm. Bars are color‐coded to reflect the direction of pathway activation or inhibition based on IPA *z*‐scores: blue indicates activation (positive *z*‐score), red indicates inhibition (negative *z*‐score), and green indicates no prediction (*z*‐score not available or near zero). The dashed vertical line indicates the FDR significance threshold (*p* < 0.05). A complete list of all enriched pathways identified by IPA are provided in the Supporting Information file.

Using the selected 181 proteins, we conducted Ingenuity Pathway Analysis (IPA) to identify canonical pathways and biological processes associated with the LPAI. The analysis revealed significant enrichment in pathways including extracellular matrix (ECM) degradation and organization, regulation of insulin‐like growth factor (IGF) transport, post‐translational protein phosphorylation, and chromatin organization (Figure 4). These pathways collectively point to alterations in structural integrity, metabolic regulation, and gene expression processes that accompany biological aging. We also assessed how these proteins related to mortality risk in the ARIC cohort using measurements at Visit 5 and found that 106 proteins showed significant associations with all‐cause mortality after adjustment for age and sex (FDR < 0.05; Figure S6), of which 89 were negatively and 92 were positively associated with mortality.

### 2.5 LPAI Is Strongly Associated With All‐Cause, Cancer and CVD Mortality Risk

**FIGURE 5:**
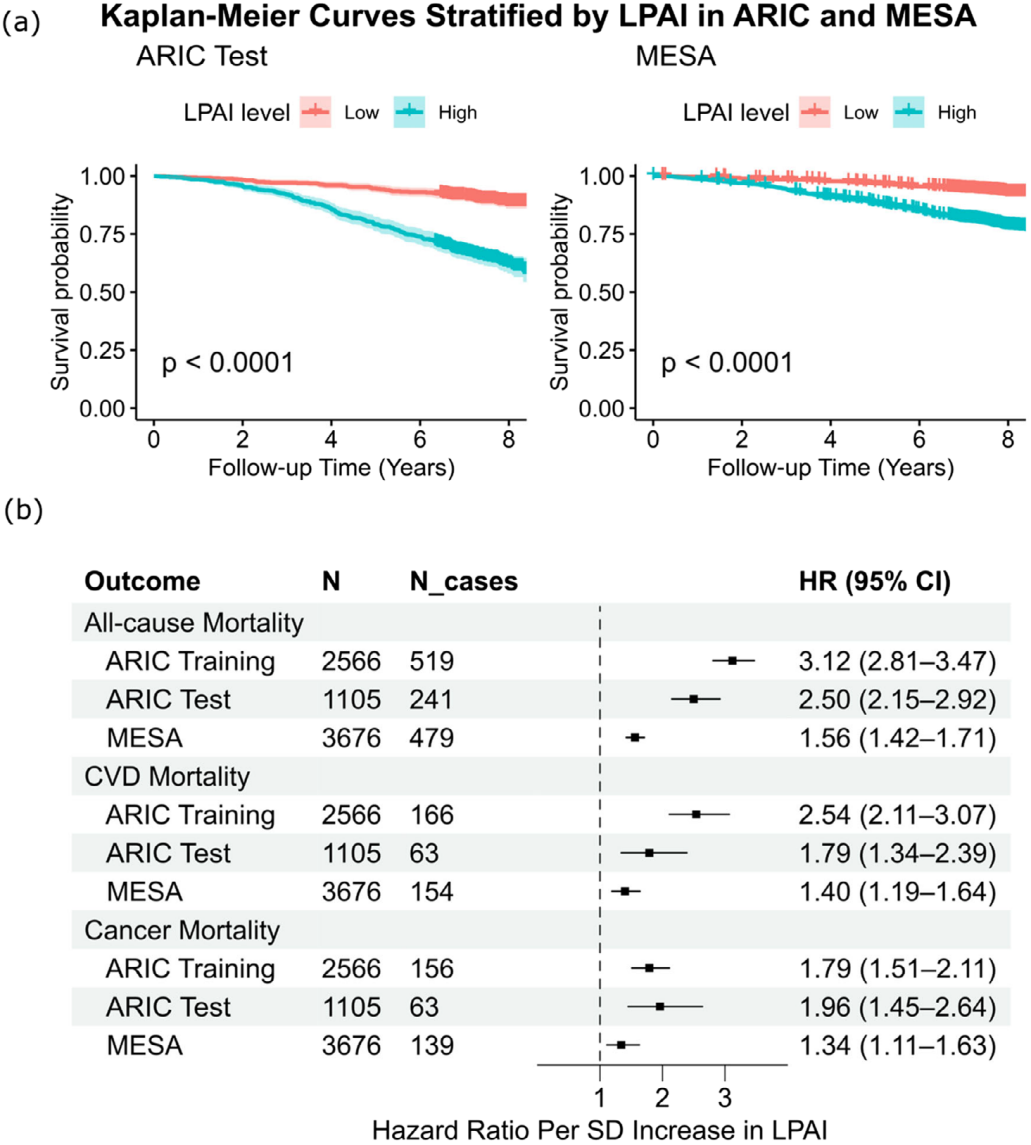
Associations of LPAI with all‐cause, cancer, and CVD mortality risk across ARIC and MESA cohorts. (a) Kaplan–Meier survival curves stratified by high versus low LPAI (dichotomized at the median) in ARIC test set and MESA. Participants with higher LPAI scores exhibited significantly lower survival probability over time (*p* < 0.0001, log‐rank test). (b) Hazard ratios and 95% confidence intervals for associations between LPAI and the risk of all‐cause, cancer and CVD mortality. Estimates were obtained using Cox proportional hazards regression models, with competing risks models (Fine and Gray method) for CVD and cancer mortality. Model adjusted for Visit/Exam 5 covariates: chronological age, sex, race, education level, study center, smoking status, alcohol use, body mass index, hypertension, diabetes, eGFR, and physical activity. *N* indicates the number of participants included in each analysis with complete data; N_cases represents the number of deaths observed during follow‐up. HR, hazard ratio; CI, confidence interval; CVD, cardiovascular disease; eGFR, estimated glomerular filtration rate.

Kaplan–Meier survival curves for all‐cause mortality showed clear separation between high‐ and low‐risk groups when LPAI was dichotomized at the cohort‐specific median (Figure 5a). In both ARIC and MESA, participants with above‐median LPAI values had significantly higher mortality risk over time compared to those below the median. In the ARIC test set, LPAI was significantly associated with all‐cause mortality (HR [95% CI] = 2.50 [2.15, 2.92] per SD), CVD mortality (HR [95% CI] = 1.79 [1.34, 2.39] per SD), and cancer mortality (HR [95% CI] =1.96 [1.45, 2.64] per SD). Statistically significant associations were also observed in MESA, where LPAI remained strongly predictive of all‐cause mortality (HR [95% CI] = 1.56 [1.42, 1.71] per SD), CVD mortality [HR (95% CI) = 1.40 (1.19, 1.64) per SD], and cancer mortality (HR [95% CI] = 1.34 [1.11, 1.63] per SD). All models were adjusted for chronological age, sex, race, study center, education, body mass index (BMI), smoking status, alcohol intake, physical activity, diabetes, hypertension, and estimated glomerular filtration rate (eGFR), which are recognized as confounders for mortality risk. These results are summarized in Table 2, Figure 5b.

**TABLE 2:** Hazard ratios (HR) of LPAI for all‐cause, cardiovascular (CVD), and cancer mortality.

Outcome	ARIC training set				ARIC test set				MESA			
	Median FU (years)	*N* (N Cases)	HR (95% CI)^a^ Per SD	*p* value	Median FU (years)	*N* (N Cases)	HR (95% CI)^a^ Per SD	*p* value	Median FU (years)	*N* (N Cases)	HR (95% CI)^a^ Per SD	*p* value
All‐cause mortality	7.47	2566 (519)	3.12 (2.81, 3.47)	< 0.0001	7.42	1105 (241)	2.50 (2.15, 2.92)	< 0.0001	7.76	3676 (479)	1.56 (1.42, 1.71)	< 0.0001
CVD mortality	7.47	2566 (166)	2.54 (2.11, 3.07)	< 0.0001	7.42	1105 (63)	1.79 (1.34, 2.39)	< 0.0001	7.76	3676 (154)	1.40 (1.19, 1.64)	< 0.0001
Cancer Mortality	7.47	2566 (156)	1.79 (1.51, 2.11)	< 0.0001	7.42	1105 (63)	1.96 (1.45, 2.64)	< 0.0001	7.76	3676 (139)	1.34 (1.11, 1.63)	0.0029

Abbreviations: FU, follow‐up; HR, hazard ratio; *N*, number of participants with complete data for each outcome and all model covariates; *N* cases, number of death cases observed during follow‐up; SD, standard deviation.

^a^ Adjusted for Visit/Exam 5 covariates: chronological age, sex, race, education level, study center, smoking status, alcohol use, body mass index, hypertension, diabetes, eGFR, and physical activity.

### 2.6 LPAI Predicts Multimorbidity and Frailty

To assess whether LPAI reflects overall aging‐related health conditions, we examined its associations with a multimorbidity index and frailty in the ARIC cohort. The multimorbidity index was derived as a weighted count of multiple concurrent chronic conditions (ranged from 0 to 10, see Section 4). Higher LPAI was significantly associated with increased multimorbidity (RR [95% CI] = 1.38 [1.31–1.45] per SD; Table S2) in the ARIC test set. Each SD increase in LPAI corresponded to a 38% higher expected multimorbidity burden. Higher LPAI was also significantly associated with greater frailty severity at Visit 5 (OR [95% CI] = 1.45 [1.23–1.70] per SD; Table S3), indicating 45% higher cumulative odds of belonging to a more severe frailty category.

### 2.7 LPAI Differs From Cross‐Sectional Proteomic Aging Clocks in Correlation Patterns

To compare with traditional cross‐sectional PACs, we constructed a midlife (mean age 58.1 ± 5.7, age range 46–70) PAC using protein measures from Visit 2 and a late‐life (mean age 76.5 ± 5.3, age range 66–90) PAC using protein measures from Visit 5 in ARIC (details in Section 4). Proteomic age acceleration (PAA) quantified the difference between biologically measured age and chronological age, which isolates biological aging effects independent of an individual's actual age.

We calculated the Pearson correlation between the LPAI, chronological age at Visit 5, and both midlife and late‐life PAAs in the training and test data of ARIC (Figure 3a). LPAI was modestly correlated with chronological age in both the training and test sets (Pearson's *r* = 0.42 and 0.40, respectively), and weakly correlated with midlife PAA (Pearson's *r* = 0.18 and 0.15, respectively) but more correlated with late‐life PAA (Pearson's *r* = 0.51 and 0.50, respectively). It was also modestly correlated with late‐life PAC (Pearson's *r* = 0.66 and 0.64, respectively).

Further, to assess how the two orthogonal FPC components relate to PAA and chronological age, we decomposed LPAI into its FPC1 and FPC2 contributions, with each contribution calculated as the sum of the corresponding FPC scores weighted by their Cox coefficients (Figure S1). The FPC1 component showed weak correlations with chronological age in both the training (*r* = 0.17) and test (*r* = 0.12) sets, whereas the FPC2 component exhibited stronger positive correlations (*r* = 0.51 and 0.55, respectively). In contrast, FPC1 was more strongly associated with PAA than FPC2, suggesting that FPC1 is more aligned with the cumulative biological aging burden largely independent of chronological age.

### 2.8 LPAI Versus Cross‐Sectional Proteomic Aging Clocks in Predicting All‐Cause Mortality, Frailty and Multimorbidity

To assess whether LPAI captures more aging‐related information than previous cross‐sectional aging clocks, we separately compared their hazard ratios for all‐cause mortality. Cox proportional hazards models were conducted independently for midlife PAA and late‐life PAA, each adjusted for the same covariates, with follow‐up starting at visit 5 for ARIC and exam 5 for MESA. Both variables were standardized to allow for meaningful comparisons of the resulting hazard ratios.

**FIGURE 6:**
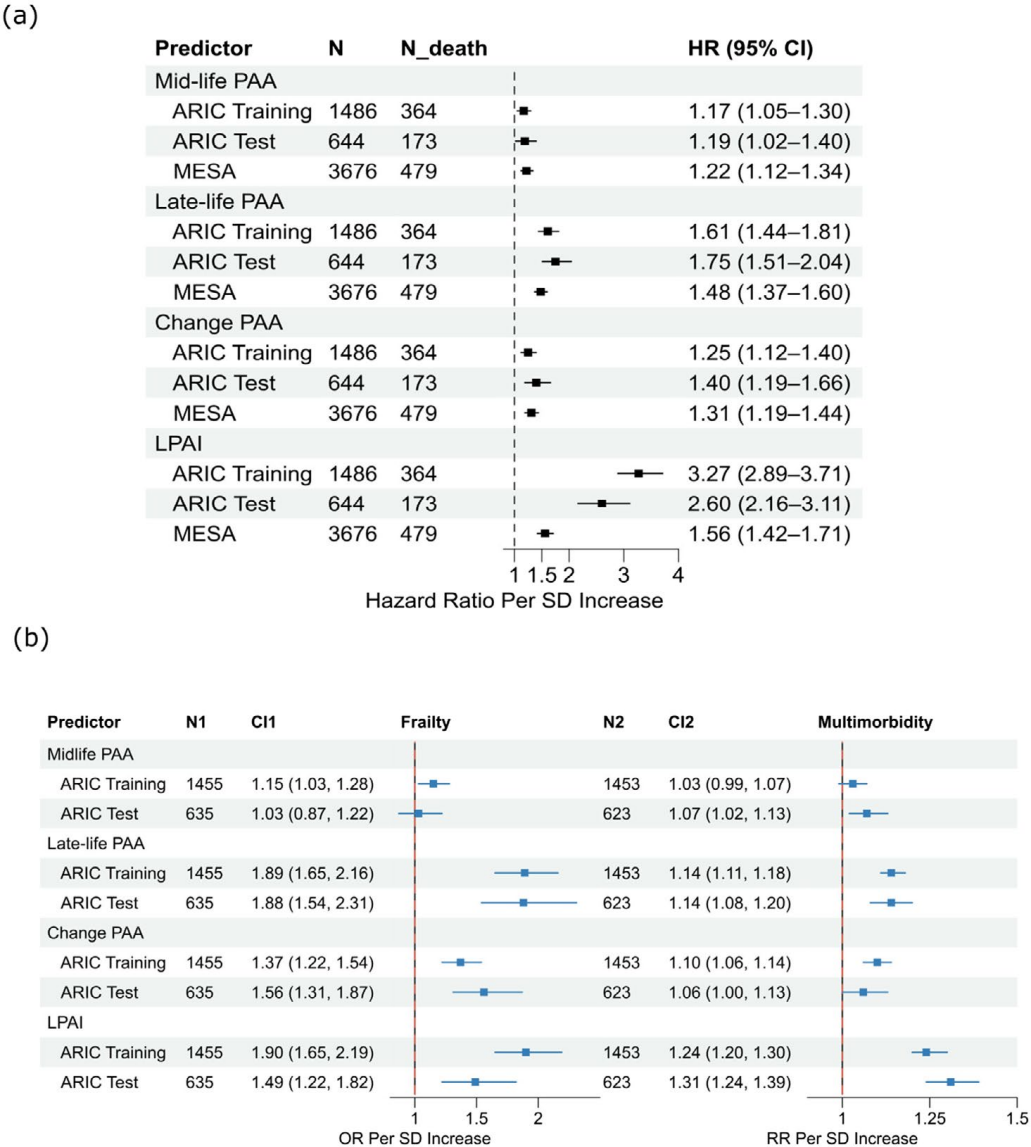
Comparison of PAAs and LPAI in relation to all‐cause mortality, frailty, and multimorbidity. (a) Hazard ratios and 95% confidence intervals per 1 SD increase in midlife PAA, late‐life PAA, change PAA (late‐life PAA minus midlife PAA), and LPAI for all‐cause mortality in ARIC and MESA. The association in ARIC was examined among participants that were not part of the training set for either the midlife or late‐life PAC. N indicates the number of participants with complete data for the outcome and all model covariates; N_death indicates the number of deaths. Model adjusted for Visit/Exam 5 covariates: chronological age, sex, race, education level, study center, smoking status, alcohol use, body mass index, hypertension, diabetes, eGFR, and physical activity. (b) Odds ratios or hazard ratios and 95% confidence intervals per 1 SD increase for frailty or multimorbidity in ARIC. Associations were examined among participants not included in the training set for either the midlife or late‐life PAC. N1/N2 indicates the number of participants with complete data for the outcome and all model covariates. Frailty models were adjusted for Visit 5 covariates: chronological age, sex, race, education level, study center, smoking status, alcohol consumption, body mass index, hypertension, diabetes, eGFR, and physical activity. Multimorbidity models were adjusted for the same covariates, excluding hypertension, diabetes, eGFR, and physical activity. CI, confidence interval; HR, hazard ratio; OR, odds ratio; RR, risk ratio; PAA, proteomic age acceleration; SD, standard deviation.

Forest plot (Figure 6a) shows that LPAI remained the strongest predictor of all‐cause mortality. Late‐life PAA was also significantly associated with mortality, while mid‐life PAA only showed a modest association in the ARIC test set. In MESA, a similar pattern was observed. Additionally, we calculated “Change PAA” by subtracting midlife PAA from late‐life PAA to assess the acceleration of proteomic aging over time. In both ARIC and MESA, its association with mortality fell between that of late‐life and midlife PAA.

Further, to assess whether LPAI improved all‐cause mortality prediction, we calculated Harrell's C‐index across different models (Figure S3). In both the ARIC test set and MESA, the model including chronological age and LPAI alone outperformed the model combining age with midlife and late‐life PAAs, with C‐indices of 0.75 versus 0.71 in ARIC and 0.79 versus 0.78 in MESA.

Beyond mortality, LPAI demonstrated the strongest association with multimorbidity, outperforming all cross‐sectional PAAs in the ARIC test set (Figure 6b). For frailty, late‐life PAA showed the strongest association in the ARIC test set. Notably, although LPAI showed a similar association with frailty as late‐life PAA in the ARIC training set, this association was attenuated in the test set.

### 2.9 LPAI Versus PhenoAge in Predicting All‐Cause Mortality, Frailty, and Multimorbidity

We retrained PhenoAge (Liu et al. 2019) in the same ARIC training set as LPAI using identical model specifications (details in Supporting Information) and evaluated its associations with all‐cause mortality, frailty, and multimorbidity in both the ARIC training and test sets.

LPAI demonstrated a significantly stronger association with all‐cause mortality compared to Phenotypic Age Acceleration (PhenoAgeAccel). In the ARIC test set, the hazard ratio per standard deviation (SD) increase for all‐cause mortality was 2.49 (95% CI: 2.13–2.91) for LPAI, compared with 1.60 (95% CI: 1.36–1.87) for PhenoAgeAccel. For frailty, LPAI also showed a stronger association, with an odds ratio per SD increase of 1.46 (95% CI: 1.25–1.72) versus 1.29 (95% CI: 1.11–1.50) for PhenoAgeAccel. The relative risks for multimorbidity were comparable between the two measures. Detailed results are presented in Tables S4–S6.

### 2.10 Sensitivity Analysis Adjusting for Batch Effects in Protein Measurements Across Visits

Because plasma samples in ARIC and MESA were collected years apart and assayed in separate batches, potential batch effects could have influenced associations. After applying the ComBat method to adjust for systematic differences across visits, the hazard ratios for all‐cause, cancer, and CVD mortality in both the ARIC test set and MESA cohort were consistent with pre‐correction estimates (Figure S4, Table S7). These results confirm that the observed associations were primarily driven by biological aging processes rather than batch effects.

### 2.11 Landmark Analysis of All‐Cause Mortality in ARIC to Assess Reverse Causality

To further evaluate the potential influence of reverse causality, we conducted landmark analysis (Rizopoulos et al. 2017) for all‐cause mortality in the ARIC cohort, redefining the start of follow‐up at 1, 3, and 5 years after Visit 5. Across all models, the association between LPAI and all‐cause mortality remained stable (HR = 2.44 [95% CI 2.08–2.85], 2.47 [2.05–2.98], and 2.07 [1.61–2.68], respectively; Table S8). The attenuated effect size observed at the 5‐year landmark likely reflects the smaller number of participants and events available for analysis, rather than a weakening of the underlying association.

## 3 Discussion

We developed a novel Longitudinal Proteomic Aging Index (LPAI) that demonstrated a proof of concept by integrating protein measurements across multiple time points to develop a metric for quantifying the trajectory of biological aging. LPAI was developed using data from 2954 participants and tested on an additional 1267 participants in ARIC, a large, prospective, population‐based cohort of White and Black individuals. Additionally, we validated this new biological aging measure in the MESA study, which included 3726 White, Black, Hispanic, and Chinese individuals, underscoring the applicability of LPAI in multi‐ethnic populations. This supports the generalizability of our method and the ease of implementation of the LPAI construction in external datasets.

We systematically evaluated the associations of LPAI with key aging‐related outcomes—mortality, multimorbidity, and frailty—which are hallmark consequences of biological aging. LPAI consistently demonstrated robust associations with all‐cause, cardiovascular, and cancer mortality across both the ARIC and MESA cohorts. In addition to mortality risk, higher LPAI was significantly associated with increased multimorbidity burden and frailty, further underscoring its utility as a robust biomarker of aging‐related health status. Proteins contributing to the LPAI were enriched in canonical pathways related to cellular and matrix structure, cell growth and metabolism, and gene regulation, all of which align with established hallmarks of aging. These pathways represent key biological processes underlying aging, including structural and mechanical decline, altered nutrient sensing and metabolic regulation, and dysregulation of gene expression (Kroemer et al. 2025).

To address potential reverse causality, we further conducted landmark analyses redefining the start of follow‐up at 1, 3, and 5 years after baseline in ARIC. The associations between LPAI and all‐cause mortality remained consistent across these analyses, indicating that the observed relationships were not primarily driven by pre‐existing illness or short‐term mortality risk.

LPAI demonstrated stronger associations with all‐cause mortality and multimorbidity than previously established cross‐sectional ARIC PACs when evaluated over identical follow‐up periods. LPAI also outperformed models incorporating both midlife and late‐life PAA in predicting all‐cause mortality, as measured by the C‐index. In ARIC, the midlife PAC was derived about 20 years before the late‐life PAC, and in MESA about 10 years earlier. As expected, the late‐life PAC, being temporally closer to mortality, showed stronger associations, while the midlife PAC remained predictive though attenuated. When compared with PhenoAge, another widely used blood biomarker–based biological aging measure also trained on mortality outcomes, LPAI showed stronger associations with mortality and frailty and comparable performance for multimorbidity. These results highlight the advantage of LPAI as a longitudinal measure derived from repeated proteomic data, rather than a single static measurement, enabling it to capture richer information on biological aging and mortality risk than cross‐sectional aging measures.

Several previously developed proteomic aging predictors provide useful points of comparison for our work. An early study trained a PAC using 1301 proteins measured by SOMAscan in a small cohort of 120 individuals and applied the model to the InCHIANTI study (Tanaka et al. 2020). They showed that PAA was associated with multimorbidity and all‐cause mortality. More recently, PACs have been developed in much larger cohorts using Olink proteomic data. For example, one study developed ProtAge in 31,808 participants from the UK Biobank Pharma Proteomics Project (UKB‐PPP) (Argentieri et al. 2024). Using LightGBM, they correlated 2897 proteins with chronological age and demonstrated that ProtAgeGap, the equivalent of PAA, predicted all‐cause mortality, multimorbidity, and 14 diseases. Another study also constructed a PAC using UKB‐PPP but trained it directly on all‐cause mortality rather than chronological age as a surrogate for biological aging (Kuo et al. 2024). Their PAC showed stronger predictive performance for mortality, COPD, pneumonia, and heart failure compared with BioAge and PhenoAge, but weaker prediction for conditions such as hypertension, stroke, and chronic kidney disease. These prior PACs can be contextualized within the generational framework established for epigenetic clocks (Crimmins et al. 2024). The first‐generation PACs (Argentieri et al. 2024; Lehallier et al. 2020; Wang et al. 2024) correlated proteins directly with chronological age, while second‐generation PACs (Kuo et al. 2024) incorporated both chronological age and proteomic data to predict mortality risk. Conceptually, LPAI aligns with second‐generation clocks in that it is trained on mortality outcomes. However, all previous PACs were constructed from cross‐sectional protein data, whereas LPAI is the first to leverage longitudinal proteomic measurements. A recent longitudinal serum proteomic study identified 86 proteins that are associated with chronological age over time (Tang et al. 2025), but their proteomic healthy aging score was still based on cross‐sectional data at baseline. By applying functional principal component analysis (FPCA), LPAI characterizes longitudinal within‐person variability in proteomic aging trajectories. Furthermore, LPAI was developed using 4684 proteins measured by the SomaScan 5K platform, a wider coverage of the human proteome than the Olink panel.

The only prior effort to develop a biological aging measure from longitudinal biomarker data is the Pace of Aging (POA) framework, where the rate of physiological decline was measured using 18 repeated biomarkers data over 12–20 years for broader population applicability. DunedinPACE, a subsequent approach, estimated precomputed POA from single‐timepoint DNA methylation data in a relatively homogeneous birth cohort of 1037 individuals in New Zealand, showing stronger or comparable associations with mortality and morbidity than earlier cross‐sectional methylation clocks such as DNAm PhenoAge and GrimAge (Belsky et al. 2022). However, DunedinPACE remains a proxy for POA using single‐timepoint methylation data and is still constrained by the predefined biomarker panel. In contrast, LPAI directly leverages repeated proteomic measurements to characterize longitudinal changes in the human proteome. LPAI was also developed in a substantially larger and more ethnically diverse sample than DunedinPACE, enhancing its generalizability.

Furthermore, most previously developed PACs and POA models assumed that aging progresses linearly, as they relied on linear modeling frameworks. In contrast, our analysis of proteomic aging trajectories identified four distinct clusters, with three showing dynamic changes across the lifespan and non‐linear inflection points around age 65, consistent with previous findings that the number of proteins showing non‐linear changes peaks near age 60 (Lehallier et al. 2019). Pathway analysis revealed concordant enrichment patterns across ARIC and MESA, with one cluster enriched for synapse‐related and nucleotide/energy‐binding pathways whose protein expression increased with age, and another enriched for blood microparticle pathways whose proteins decreased with age. In MESA, we additionally observed two‐stage non‐linear shifts in protein‐binding pathways, such as heparin and glycosaminoglycan binding. These pathways are consistent with those reported in the previous study of non‐linear proteomic aging patterns (Lehallier et al. 2019), suggesting that plasma proteomic aging follows non‐linear yet coordinated changes across biological processes. LPAI effectively captures complex non‐linear proteomic patterns through FPCA and translates them into a biological aging indicator.

By leveraging FPCA, LPAI integrates features of both PAA and POA, reflecting cumulative aging burden and dynamic changes over time. Graphical examination of the FPCs revealed two possible orthogonal dimensions of aging: FPC1 appeared more aligned with overall accumulation, reflecting parallel shifts in protein levels across age, whereas FPC2 likely represented trajectory, capturing localized changes within specific age intervals. Approximately, FPC1 contributions quantify an individual's cumulative aging burden relative to the cohort‐predicted mean, conceptually analogous to PAA from cross‐sectional measures, while FPC2 contributions reflect age‐specific accelerations or decelerations in aging pace, aligning with POA. The correlation patterns supported these interpretations. When LPAI was decomposed into FPC1 and FPC2 contributions, the FPC1 contribution showed weak correlation with chronological age but a stronger association with PAA, indicating age‐independent accumulation of biological burden. In contrast, the FPC2 contribution correlated modestly with chronological age, consistent with prior evidence of increasing aging pace across the lifespan (Belsky et al. 2022).

Although LPAI remained significantly associated with all‐cause, cancer, and CVD mortality in both the ARIC and MESA test sets, the hazard ratios were attenuated in MESA. One possible explanation is that ARIC participants were older on average at the final visit, representing a higher risk population compared to MESA (Harding et al. 2021). This interpretation is supported by the Kaplan–Meier survival curves, which show lower overall survival in ARIC. The sampling designs of the two studies also differed. The ARIC cohort was drawn through probability sampling within four U.S. communities, making it broadly representative of middle‐aged Black and White adults in those regions (Wright et al. 2021), whereas MESA recruited participants free of clinical cardiovascular disease at baseline using stratified sampling by age, sex, and race/ethnicity (Bild et al. 2002). Another explanation for the attenuated associations is the difference in time intervals between proteomic measurements. In MESA, the three protein measurements were conducted at evenly spaced intervals of approximately 5 years each, whereas in ARIC, the first two visits were 3 years apart, followed by a much longer interval of 15 years. Because FPCA was trained to capture the largest variation in protein trajectories within the ARIC training set, the resulting FPC scores may not translate optimally when applied to MESA. This is supported by the observation that estimated mean trajectories for some proteins differed between the two cohorts, suggesting that age structure and visit spacing influence the shape of derived trajectories and may dilute risk signals.

Several limitations should be noted regarding the construction of LPAI. (1) Only participants with proteomic data from all three visits in ARIC (exams in MESA) were included. Participants with missing protein measurements, potentially due to ill health or mortality prior to the second or third proteomic measurement, were excluded. Therefore, both the training and test sets likely represented a healthier subset of the population. This selection may have attenuated the observed associations between LPAI and mortality or other aging‐related outcomes. (2) Because LPAI was trained in the ARIC cohort, its generalizability may be limited by cohort‐specific age structure, visit spacing, and measurement designs, leading to potential overfitting. This may explain the attenuated associations for frailty and mortality observed in the ARIC test set and MESA. Clinically, directly applying the ARIC‐derived coefficients to other populations could dilute risk signals, whereas retraining LPAI on cohort‐specific data may improve performance across diverse populations. (3) FPCA assumes that each individual's proteomic aging follows a smooth, continuous function over time. With only three time points available, this approach may emphasize long‐term aging trends while smoothing over short‐term changes. Future studies with more frequent longitudinal proteomic measurements could capture finer‐scale biological variability in aging trajectories.

Additionally, while we compared LPAI with previously established cross‐sectional PACs built in ARIC and with PhenoAge, we were unable to evaluate other widely used biological aging measures. DunedinPACE could not be assessed because DNA methylation data are not yet available in the full ARIC and MESA cohorts. BioAge could not be replicated due to the unavailability of several required clinical biomarkers in ARIC. Recently developed PACs trained on Olink proteomic data from the UK Biobank could not be applied to our cohorts, as technical differences between the Olink and SomaScan platforms limit cross‐platform comparability and no standardized harmonization framework currently exists (Moqri et al. 2024). Future replication of LPAI in additional cohorts with multi‐omics data will enable benchmarking against other biological aging measures to further evaluate its comparative performance. To support replication and broader use, we developed an open‐source package (https://github.com/scottiejj/LPAI) that allows researchers to apply the ARIC‐trained model or retrain LPAI on their own longitudinal proteomic data, providing an opportunity to validate and extend LPAI across diverse populations and research settings.

In conclusion, we proposed a novel method that combines FPCA and Elastic Net Penalized Cox Proportional Hazards Regression to establish a new biological aging measurement, LPAI. Utilizing two large, ethnically diverse datasets from the ARIC and MESA study, we demonstrated and validated LPAI's associations with mortality, multimorbidity, and frailty. Key strengths of LPAI include (1) its ability to leverage longitudinal proteomic data to construct an aging measure that reflects health status, (2) its ease of implementation and computational efficiency when analyzing high‐dimensional proteomic data, and (3) its ability to capture non‐linear interindividual proteome variability. LPAI complements existing biological aging measures by capturing both cumulative aging burden and dynamic longitudinal changes, providing proof‐of‐concept that variation in proteomic profiles across individuals over time helps explain the relationship between aging, health, and disease.

## 4 Methods

### 4.1 Study Population

This study examined both White and Black men and women enrolled in the Atherosclerosis Risk in Communities (ARIC) Study. During the first visit (1987–1989), a total of 15,792 individuals aged 45–64 years were recruited across four study locations; participants reporting races other than White or Black (*n* = 48 at Visit 1) were excluded from this study due to insufficient sample size for reliable analysis. Details of the ARIC study can be found in our previous paper (Wang et al. 2024). Proteomic measurements were made in plasma samples collected at Visit 2 (1990–1992), Visit 3 (1993–1995), and Visit 5 (2011–2013). A total of 4221 ARIC participants had proteomic measurements across all three visits (Figure 1c).

Our external validation analyses involved individuals from the MESA study. Participants in MESA are ethnically diverse, including White, Black, Hispanic, and Chinese individuals. Details of the MESA study are available at the MESA website (MESA 2019). The MESA study includes 6814 men and women, ranging in age from 45 to 84 when the study began at the baseline level. MESA has proteomic data collected at Exam 1 (2000–2002), Exam 4 (2005–2007), and Exam 5 (2010–2012). In the MESA validation set, 3726 participants were included who had proteomic data at all three exams (Figure 1c).

### 4.2 Protein Measurement and Quality Control

Plasma samples from the ARIC study were analyzed using the SomaScan (V4.0) platform, a Slow Off‐rate Modified Aptamer (SOMAmer)‐based capture array developed by Somalogic Inc. (Boulder, CO, USA) (Gold et al. 2010; Kim et al. 2018; Rooney et al. 2022). Comprehensive details on the SomaScan assay methodology and data normalization procedures have been previously documented (Candia et al. 2022). To enhance data reliability, proteins were excluded if they exhibited a Bland–Altman coefficient of variation (CVBA) exceeding 50%, had a variance below 0.01 on the log scale, or demonstrated non‐specific binding to mouse Fc‐fusion proteins, contaminants, or non‐protein molecules (Rooney et al. 2022). Protein concentrations were measured in relative fluorescent units (RFU) and were log2‐transformed. After the exclusion process, 4955 aptamers were retained for analysis.

MESA utilized the SomaScan array (V4.1) to measure nearly 7000 aptamers in plasma samples. Of these aptamers, 4952 are common to the 4955 aptamers measured in the ARIC study.

For this analysis, we selected 4684 proteins, uniquely identified by UniProt IDs, from the 4952 shared aptamers.

### 4.3 Assessment of Mortality in ARIC and MESA

In ARIC, deaths were identified through annual (semi‐annual after 2012) follow‐up calls, hospital records, state registries, and National Death Index linkage until December 31, 2019 (Rooney et al. 2020), which is date of administrative censoring (For participants in the Jackson Center, the administrative censoring was on December 31, 2017 rather than 2019 because of difficulties obtaining records from a large hospital in the area). Mortality was classified as all‐cause, cardiovascular (ICD‐9: 390–459; ICD‐10: I00–I99), or cancer‐related (ICD‐9: 140–239; ICD‐10: C00–C97). All‐cause mortality, CVD mortality, and cancer mortality among ARIC cohort participants were assessed until December 31, 2019. Of note, in this study, the start of follow‐up for mortality was the date of Visit 5 (which is the date of the last protein measurement) until the date of mortality, censored at the loss of follow‐up or the end of follow‐up, whichever occurred first.

In MESA, participants were followed for up to 18 years until December 31, 2018, with systematic mortality and CVD events adjudication (Post et al. 2022). Cancer deaths were classified by ICD‐10 codes. In this study, the start of follow‐up for mortality was the date of Exam 5.

### 4.4 Assessment of Multimorbidity and Frailty in ARIC

Multimorbidity at ARIC Visit 5 was quantified using a weighted index derived from a prior ARIC study that adapted the Charlson Comorbidity Index (Charlson et al. 1987; Martinez‐Amezcua et al. 2021). For each participant, the index was a weighted sum of the chronic conditions present by Visit 5 (prevalent at baseline or diagnosed during follow‐up): myocardial infarction, peripheral vascular disease, heart failure, chronic obstructive pulmonary disease, chronic kidney disease, diabetes, and dementia (weight = 1 each), plus stroke and cancer (weight = 2 each). Other conditions listed in the original Charlson Comorbidity Index were not captured in ARIC and thus not included. ARIC cancer cases were identified via state cancer registries supplemented by medical records and hospital discharge codes (Joshu et al. 2017). Distribution of the multimorbidity index of the ARIC training and test set is shown in Figure S2.

Frailty at ARIC Visit 5 was assessed using the Fried frailty phenotype, which defines frailty according to five components: weight loss, exhaustion, low energy expenditure, slowness, and weakness (Fried et al. 2001). Participants were classified as frail if three or more components were present, prefrail if one or two were present, and robust if none were present with complete component data. Component definitions and thresholds were based on previously published criteria (Kucharska‐Newton et al. 2017). For analysis, frailty status was coded ordinally (1 = frail, 2 = prefrail, 3 = robust).

### 4.5 Assessment of Other Characteristics of Interest in ARIC and MESA

Other characteristics of interest included demographic and lifestyle/clinical characteristics, namely chronological age, sex, race, study center, education, smoking status, alcohol intake, body mass index (BMI), physical activity, diabetes status, hypertension status, and eGFR, which were assessed at Visit 5 for ARIC or Exam 5 for MESA (MESA 2019; Wright et al. 2021). Educational attainment was collected at Visit 1 for ARIC and at Exam 1 for MESA. Descriptive statistics for both cohorts, including distributions of quantitative variables and categories for categorical variables, are provided in Table S1.

### 4.6 Construction of LPAI in the Training Set and in the Test Sets

LPAI was trained in ARIC using a two‐step approach involving Functional Principal Component Analysis (FPCA) followed by Elastic Net Penalized Cox Proportional Hazards Regression (Figure 1b). FPCA is a statistical technique used to analyze functional data consisting of continuous functions. It identifies the most significant modes of variation within the curves, allowing for the representation of complex functional relationships with fewer dimensions using a set of eigenfunctions and Functional Principal Component (FPC) scores. The trajectory of each protein at three visits for an individual can be approximated by a continuous function of age. An additional mathematical formulation for this approximation can be found in the Supporting Information.

A random selection of 70% of all ARIC participants (*N* = 2954) formed the training set for applying FPCA to each protein, while the remaining 30% (*N* = 1267) of ARIC participants constituted the internal test set. Participants were assigned by uniform random sampling without replacement and without stratification. Subsequently, the FPC scores were concatenated and used as features for the Elastic Net Penalized Cox Proportional Hazards Regression. The time‐to‐event outcome for the Cox Regression was the time‐to‐death due to all‐cause mortality. We set the elastic net mixing coefficient *α* to 0.5, which determines the balance between L1 and L2 penalty. The regularization parameter *λ*, controlling the overall strength of sparsity, was selected using 10‐fold cross‐validation to minimize cross‐validation error. The elastic net performs feature selection on principal component scores for different proteins, helping to identify those whose variation over time significantly impacts health status, while also mitigating overfitting. Finally, LPAI was the log hazard ratio, calculated as the weighted sum of FPC score *Z*s from the trained Cox elastic net model:

$${\text{LPAI}}_{i}=\sum_{j=1}^{J}\left(\beta_{j}\cdot Z_{\mathrm{ij}}\right)$$

where ‘*J* ’ was the number of non‐zero FPCs selected by the Elastic Net Penalized Cox Proportional Hazards Regression.

After training, data from the ARIC and MESA test sets were projected onto the trained function eigenfunctions to compute FPC scores. The detailed mathematical formulation of this projection is provided in the Supporting Information. LPAI for the test sets was then calculated as a linear combination of these FPC scores, using the trained coefficients from the Cox elastic net model.

Given the limited number of measurements per individual (only three per protein) and the unequal intervals between protein measurements, the Principal Analysis by Conditional Expectation (PACE) method was employed for FPCA analysis to effectively handle sparse and irregularly sampled data. The PACE method was developed to handle sparsely and irregularly sampled random trajectories (Yao et al. 2005).

### 4.7 Construction of Midlife and Late‐Life Cross‐Sectional Proteomic Aging Clocks

In ARIC, we previously developed a midlife PAC using Visit 2 plasma proteomic data from 11,761 participants aged 46–70 and a late‐life clock using Visit 5 plasma proteomic data from 5183 participants aged 66–90 (Wang et al. 2024). At each visit, the PAC was trained on chronological age using elastic net regression in two‐thirds of healthy participants. Proteomic age acceleration was defined as the residual from regressing the PAC on chronological age. We have previously shown that a larger PAA is associated with an increased risk of mortality. In this study, we applied the ARIC midlife PAC to MESA Exam 1 data and the ARIC late‐life PAC to MESA Exam 5 data by using the protein weights derived from ARIC to construct midlife and late‐life proteomic aging clocks in MESA, respectively. Notably, in contrast to our previous study evaluating the association of PAA with all‐cause mortality in ARIC, this study excluded participants who had been included in either the midlife or late‐life PAC training sets, and for all analyses, follow‐up began at Visit 5 in ARIC or Exam 5 in MESA to allow direct comparison with LPAI.

### 4.8 Protein Trajectory Clustering

To visualize trajectories of each protein across age, we applied locally estimated scatterplot smoothing (LOESS), a nonparametric regression that fits smooth curves to z‐scored protein expression levels as a function of age. We then applied k‐means clustering, an unsupervised algorithm that clusters data by minimizing within‐cluster variation, to the fitted curves to identify proteins with similar age‐related trajectories. The optimal number of clusters for k‐means was selected using the elbow method, which determines the point beyond which additional clusters provide minimal reduction in within‐cluster variance.

### 4.9 Pathway Enrichment Analysis

To interpret the biological implication of the four protein trajectory clusters obtained from LOESS, we performed pathway enrichment analysis using Gene Ontology (GO) annotations implemented in the ToppGeneSuite (https://toppgene.cchmc.org/) (Chen et al. 2009). The 4684 measured proteins in ARIC served as the reference background set. The top enriched pathways for each cluster are provided in the Supporting Information.

For proteins selected in the LPAI model, we further performed Ingenuity Pathway Analysis (IPA, QIAGEN Inc.) using the same 4684 ARIC proteins as the background reference. Enrichment significances were determined based on the right‐tailed Fisher's exact test with FDR correction at *p* < 0.05.

### 4.10 Sensitivity Analysis for Batch Effects

Plasma samples in ARIC and MESA were collected several years apart and assayed in separate batches, which could introduce potential batch effects. We applied the ComBat (Johnson et al. 2007) algorithm implemented in the R sva package (version 3.54.0) (Leek et al. 2024), adjusting for chronological age, separately in ARIC and MESA to correct batch‐related variation in protein expression. LPAI was then retrained and tested using the same sets of individuals after batch correction.

### 4.11 Statistical Analysis

All analyses were performed using R (version 4.3.1). The PACE algorithm for performing FPCA was executed using the fdapace package (version 0.6.0) (Zhou et al. 2024), and Elastic Net Penalized Cox Proportional Hazards Regression was conducted using the glmnet package (version 4.1.8) (Simon et al. 2011). The resulting LPAI was standardized (mean = 0, standard deviation = 1) and treated as a continuous variable in all downstream analyses. For visualization and descriptive purposes, LPAI was additionally dichotomized at the cohort‐specific median into high and low groups. All primary association and predictive analyses were conducted using continuous, standardized LPAI values.

Cox proportional hazards regression was used to calculate hazard ratios (HRs) and 95% confidence intervals (CIs) for mortality from all‐cause, cancer, and CVD in relation to LPAI. For the associations with CVD and cancer mortality, deaths from other causes were treated as competing events using the Fine and Gray method. All analyses were conducted using the survival package (version 3.8‐3) (Therneau 2024) in R (version 4.3.1). Associations between LPAI and multimorbidity were evaluated using Poisson regression via the stats package, with the multimorbidity index modeled as a count outcome. Associations between LPAI and frailty were assessed using ordered logistic regression, implemented via the MASS package (Venables and Ripley 2002) (version 7.3‐65), with frailty modeled as a three‐level ordinal outcome (1 = robust, 2 = pre‐frail, 3 = frail). To evaluate predictive performance for all‐cause mortality, we used Harrell's C‐index, which quantifies the proportion of correctly ordered risk predictions (Harrell Jr. et al. 1996). Associations between individual protein levels and all‐cause mortality were evaluated using age and sex‐adjusted Cox models with FDR correction for multiple testing.

To assess the potential influence of reverse causality, we conducted landmark analysis (Rizopoulos et al. 2017) for all‐cause mortality in ARIC, redefining the start of follow‐up at 1, 3, and 5 years after baseline (Visit 5 in ARIC). Landmark analysis limits the risk set to participants who remain event‐free up to each specified time point, thereby reducing potential effects from pre‐existing conditions. Participants who died before the respective landmark were excluded, and Cox proportional hazards models were re‐estimated using delayed entry at the corresponding landmark time.

## Author Contributions

Zexi Rao and Weihua Guan conceived and designed the study. Zexi Rao performed statistical analysis and drafted the manuscript. Shuo Wang and Anna Prizment facilitated data access and provided cohort expertise. Weihua Guan and Anna Prizment acquired funding and served as corresponding authors. All authors reviewed and approved the final manuscript.

## Funding

This work was supported by the National Center for Advancing Translational Sciences, 1UM1TR004405. National Cancer Institute, R01CA267977. National Institute on Aging, R21AG079242. National Heart, Lung, and Blood Institute, R01HL159081.

## Conflicts of Interest

The authors declare no conflicts of interest.

## Supporting information


**Appendix S1:** acel70317‐sup‐0001‐AppendixS1.docx.

**Appendix S2:** acel70317‐sup‐0002‐AppendixS2.xlsx.

**Appendix S3:** acel70317‐sup‐0003‐AppendixS3.xlsx.

## Data Availability

ARIC: ARIC data used in this study can be accessed in accordance with the following policy: https://aric.cscc.unc.edu/aric9/sites/default/files/publications/policies/ARIC%20data%20sharing.pdf. Additionally, ARIC data are available through BioLINCC (a controlled access database). MESA: MESA data used in this study are available through the Multi‐Ethnic Study of Atherosclerosis (MESA) Data Coordinating Center. Researchers can request access via the MESA website (https://www.mesa‐nhlbi.org). For further details, inquiries can be directed to the corresponding author upon request. Code to calculate LPAI: Publicly available on GitHub at https://github.com/scottiejj/LPAI.
