# Supplementary material for: A Novel Longitudinal Proteomic Aging Index Predicts Mortality, Multimorbidity, and Frailty in Older Adults
Source: Aging Cell. 2025 Dec 8;25(1):e70317. doi: 10.1111/acel.70317 (PMC12741248; doi:10.1111/acel.70317)
Supplement: Supplementary file 1 — Appendix S1: acel70317‐sup‐0001‐AppendixS1.docx. [file ACEL-25-e70317-s002.docx]

**Supplementary Material**

**Mathematical Formulation for FPCA score approximation**

The estimated curve of a protein for individual $i$ is given by:

$$X_{i}\left( t \right)=\mu\left( t \right)+\sum_{k=1}^{K} \left( Z_{ik}\phi_{k}\left( t \right) \right)$$

where $K$ is the number of eigenfunctions used to approximate the curves, determined by the percentage of variance explained. The function $\mu(t)$ denotes the mean trajectory of the protein over time, $\phi_{k}\left( t \right)$ is the $k$-th eigenfunction, and $Z_{ik}$ is the $k$-th functional principal component (FPC) score for the $i$-th individual. This formulation allows the aging trajectory of a protein to be represented as a sum of time-varying eigenfunctions weighted by individual-specific scores.

For a new individual m in the test set, the k-th FPC score can be estimated by numerical integration:

$$\xi_{mk}=\int\left( X_{m}\left( t \right)-\mu\left( t \right) \right)\cdot\phi_{k}\left( t \right)dt$$

, where $\mu\left( t \right)$ and $\phi_{k}\left( t \right)$ are estimated from FPCA in the training set.

When each individual has only a few irregularly spaced observations, the sparse version of FPCA can be applied using the **Principal Analysis by Conditional Estimation(Yao et al., 2005)** algorithm. In this framework, the mean function $\mu\left( t \right)$ and covariance surface $C\left( s,t \right)$ are estimated using local smoothing across all individuals. The eigenfunctions $\phi_{k}\left( t \right)$are then obtained through eigendecomposition of the smoothed covariance surface. For each individual, the k-th FPC scoreis estimated as the conditional expectation given the observed values:

$$\xi_{ik}=E\left[ \xi_{ik} | Y_{i} \right]=\lambda_{k}\phi_{k}\left( t_{new} \right)^{T}\left[ C_{t_{new}}+\sigma^{2}I \right]^{-1}[Y_{i}-\mu\left( t_{new} \right)]$$

Here, $Y_{i}$ is the vector of observed protein measurements for individual i at time points $t_{new}$, and $\sigma^{2}$ is the estimated measurement error variance. This approach allows accurate estimation of FPC scores even with sparse, noisy data. The resulting FPC scores summarize individual-level trajectory features and can be directly used as predictors in downstream regularized Cox regression.

**Log partial Likelihood of the Elastic Net Penalized Cox Proportional Hazards Regression**

$$l\left( \boldsymbol{\beta} \right)=\sum_{i=1}^{m} \left( \mathbf{x}_{j\left( i \right)}^{\top}\boldsymbol{\beta}-log\left( \sum_{j\in\mathcal{R}_{i}} e^{\mathbf{x}_{j}^{\top}\boldsymbol{\beta}} \right) \right)$$

where $\mathbf{x}_{j}$ is a $P\times1$ vector of the FPC scores (with $P=K\times J$, where K is the number of principal components per protein and J is the number of proteins), $\boldsymbol{\beta}$ is the vector of regression coefficients. A penalty is added when maximizing the scaled log partial likelihood:

$$\lambda P_{\alpha}\left( \boldsymbol{\beta} \right)=\lambda\left( \alpha\sum_{i=1}^{p} \left| \beta_{i} \right|+\frac{1}{2}\left( 1-\alpha\right)\sum_{i=1}^{p} \beta_{i}^{2} \right)$$

$\lambda$ is the regularization parameter controlling the overall penalty strength, $\alpha\in\left[ 0,1 \right]$ balances between L1 (lasso) and L2 (ridge) regularization.

**Training of PhenoAge and Calculation of PhenoAge Acceleration**

We retrained PhenoAge using the same ARIC training set as LPAI with the publicly available BioAge package(Kwon & Belsky, 2021) on GitHub. Participants with complete data for the nine clinical biomarkers measured at ARIC Visit 5 included in the original PhenoAge model (Liu et al., 2019)(albumin, creatinine, glucose, C-reactive protein, lymphocyte percentage, mean corpuscular volume, red cell distribution width, alkaline phosphatase, and white blood cell count) were included during training (N = 2796). For albumin and alkaline phosphatase, we used measurements from the SomaScan panel, which differ in units from the original PhenoAge specification (relative fluorescence units rather than g/dL for albumin and U/L for alkaline phosphatase). All remaining biomarkers were derived from standard clinical assays.

The nine biomarkers and chronological age at Visit 5 were used to fit a Cox proportional hazards model for all-cause mortality, with follow-up beginning at Visit 5. Phenotypic Age Acceleration (PhenoAgeAccel) was calculated as the residual from regressing PhenoAge on chronological age, representing the deviation of biological age from chronological age.

References:

Kwon, D., & Belsky, D. W. (2021). A toolkit for quantification of biological age from blood chemistry and organ function test data: BioAge. GeroScience, 43(6), 2795–2808. https://doi.org/10.1007/s11357-021-00480-5

Liu, Z., Kuo, P.-L., Horvath, S., Crimmins, E., Ferrucci, L., & Levine, M. (2019). A new aging measure captures morbidity and mortality risk across diverse subpopulations from NHANES IV: A cohort study. PLOS Medicine, 15(12), e1002718-. https://doi.org/10.1371/journal.pmed.1002718

Yao, F., Müller, H.-G., & Wang, J.-L. (2005). Functional Data Analysis for Sparse Longitudinal Data. Journal of the American Statistical Association, 100, 577–590. https://doi.org/10.1198/016214504000001745

|  | **ARIC Training**  **sett 5** | **ARIC Test Set** | **MESA Validation Set** |
| --- | --- | --- | --- |
| **N** | 2954 | 1267 | 3726 |
| **Mean Age at Visit 5 in ARIC and Exam 5 in MESA (SD)** | 76.2 (5.2) | 76.1(5.1) | 70.1(8.9) |
| White, % | 83.8 | 83.2 | 41.5 |
| Black, % | 16.2 | 16.8 | 25.5 |
| Chinese, % | NA | NA | 11.3 |
| Hispanic, % | NA | NA | 21.7 |
| **Female, %** | 57.5 | 59.0 | 52.4 |
| **Education Level** |  | | |
| Less than high school graduate, % | 12.8 | 12.1 | 14.2 |
| High school equivalent, % | 43.0 | 44.6 | 17.8 |
| At least some college, % | 44.3 | 43.3 | 68.0 |
| **Smoking Status** |  | | |
| Current Smoker, % | 6.2 | 5.5 | 7.6 |
| Former Smoker, % | 51.3 | 52.2 | 48.4 |
| Never Smoker, % | 42.5 | 42.3 | 44.0 |
| **Alcohol Consumption Status** |  | | |
| Current Drinker, % | 51.0 | 53.5 | 44.0 |
| Former Drinker, % | 29.5 | 25.4 | 38.0 |
| Never Drinker, % | 19.5 | 21.1 | 18.1 |
| **Diabetes, %** | 31.2 | 33.9 | 41.5 |
| **Physical Activity (SD) ^1^** | 2.6(0.8) | 2.6(0.8) | 5106.2(5873.7) |
| **Hypertension, %** | 72.7 | 73.6 | 60.0 |
| **Mean BMI, *kg/m*^2^ (SD)** | 28.6(5.6) | 28.6(5.1) | 28.6(5.6) |
| **Mean eGFR, mL/min/1.73 m2 (SD)** | 71.9(17.4) | 71.9(16.6) | 66.9(15.0) |

SD – standard deviation; BMI – body mass index; eGFR – estimated glomerular filtration rate.

^1^ ARIC: Physical activity was assessed as a sport index during leisure time, ranged from 1 to 5. MESA: Physical activity defined by Weekly MET-minutes, Unit: MET-MIN/WK M-SU

Supplementary Table 1. Characteristics of ARIC and MESA participants at Visit/Exam 5.

|  | **ARIC Training Set (N=** **2544)** | | **ARIC Test Set (N=** **1087)** | |
| --- | --- | --- | --- | --- |
|  | **RR (95% CI)** ^1^  **Per SD** | **P value** | **RR (95% CI)** ^1^  **Per SD** | **P value** |
| Multimorbidity at Visit 5 | 1.33(1.29, 1.37) | <0.0001 | 1.37(1.31, 1.48) | <0.0001 |

Supplementary Table 2: Association Between LPAI and Multimorbidity Index in the ARIC Cohort: Risk Ratios

^1^Adjusted for Visit 5 covariates: chronological age, gender, race, education level, study center, smoking status, alcohol use, body mass index. N reflects number of participants with complete data for outcome and all model covariates.

|  | **ARIC Training Set (N=2517)** | | **ARIC Test Set (N=1090)** | |
| --- | --- | --- | --- | --- |
|  | **OR (95% CI)** ^1^  **Per SD** | **P value** | **OR (95% CI)** ^1^  **Per SD** | **P value** |
| Frailty at Visit 5 | 1.79(1.60, 1.99) | <0.0001 | 1.45(1.23, 1.70) | <0.0001 |

Supplementary Table 3: Association Between LPAI and Frailty in the ARIC Cohort: Odds Ratios

^1^Adjusted for Visit 5 covariates: chronological age, gender, race, education level, study center, smoking status, alcohol use, body mass index, hypertension, diabetes, eGFR, and physical activity. N reflects number of participants with complete data for outcome and all model covariates.

|  | **ARIC Training Set (N=2519)** | | **ARIC Test Set (N=1082)** | |
| --- | --- | --- | --- | --- |
|  | **HR (95% CI)** ^1^  **Per SD** | **P value** | **HR (95% CI)** ^1^  **Per SD** | **P value** |
| PhenoAgeAccel | 1.55(1.42, 1.68) | <0.0001 | 1.60(1.36, 1.87) | <0.0001 |
| LPAI | 3.12(2.81, 3.48) | <0.0001 | 2.49(2.13, 2.91) | <0.0001 |

Supplementary Table 4: Hazard ratios of PhenoAgeAccel and LPAI for all-cause mortality.

¹Models were adjusted for Visit 5 covariates, including chronological age, sex, race, education level, study center, smoking status, alcohol use, body mass index, hypertension, diabetes, eGFR and physical activity. N denotes the number of participants with complete data for the outcome and all model covariates.

|  | **ARIC Training Set (N=** **2499)** | | **ARIC Test Set (N=** **1064)** | |
| --- | --- | --- | --- | --- |
|  | **RR (95% CI)** ^1^  **Per SD** | **P value** | **RR (95% CI)** ^1^  **Per SD** | **P value** |
| PhenoAgeAccel | 1.21(1.18, 1.25) | <0.0001 | 1.30(1.24, 1.36) | <0.0001 |
| LPAI | 1.33(1.29, 1.38) | <0.0001 | 1.37(1.31, 1.45) | <0.0001 |

Supplementary Table 5: Relative risks of PhenoAgeAccel and LPAI for multimorbidity.

¹Models adjusted for Visit 5 covariates: chronological age, gender, race, education level, study center, smoking status, alcohol use, body mass index. N reflects number of participants with complete data for outcome and all model covariates.

|  | **ARIC Training Set (N=2475)** | | **ARIC Test Set (N=** **1069)** | |
| --- | --- | --- | --- | --- |
|  | **OR (95% CI)** ^1^  **Per SD** | **P value** | **OR (95% CI)** ^1^  **Per SD** | **P value** |
| PhenoAgeAccel | 1.24(1.13, 1.37) | <0.0001 | 1.29(1.11, 1.50) | <0.0001 |
| LPAI | 1.76(1.58, 1.97) | <0.0001 | 1.46(1.25, 1.72) | <0.0001 |

Supplementary Table 6: Odds ratios of PhenoAgeAccel and LPAI for frailty.

¹Models were adjusted for Visit 5 covariates, including chronological age, sex, race, education level, study center, smoking status, alcohol use, body mass index, hypertension, diabetes, eGFR and physical activity. N denotes the number of participants with complete data for the outcome and all model covariates.

|  | **ARIC Training Set** | | **ARIC Test Set** | | **MESA** | |
| --- | --- | --- | --- | --- | --- | --- |
|  | **N Cases** | **HR (95% CI)**^1^ **Per SD** | **N Cases** | **HR (95% CI)**^1^ **Per SD** | **N Cases** | **HR (95% CI)**^1^ **Per SD** |
| All-cause Mortality | 519 | 2.49 (2.27, 2.73) | 241 | 2.26 (1.98, 2.58) | 479 | 1.64 (1.50, 1.79) |
| CVD Mortality | 166 | 2.16(1.84, 2.52) | 63 | 1.51 (1.19, 1.90) | 154 | 1.42 (1.21, 1.68) |
| Cancer mortality | 156 | 1.71(1.49, 1.97) | 63 | 2.04 (1.64, 2.53) | 139 | 1.42 (1.20, 1.69) |

Supplementary Table 7: Hazard Ratios of LPAI for All-Cause, Cardiovascular, and Cancer Mortality After ComBat Adjustment

^1^Adjusted for Visit/Exam 5 covariates: chronological age, gender, race, education level, study center, smoking status, alcohol use, body mass index, hypertension, diabetes, eGFR, and physical activity. N represents number of participants with complete data for each outcome and all model covariates; N cases represents the number of deaths observed during follow-up

| Landmark Start (Years After Visit 5) | **ARIC Training Set (N=2475)** | | | **ARIC Test Set (N=** **1069)** | | |
| --- | --- | --- | --- | --- | --- | --- |
|  | **Participants at Risk, N** | **N Cases** | **HR (95% CI)** ^1^  **Per SD** | **Participants at Risk, n** | **N Cases** | **HR (95% CI)** ^1^  **Per SD** |
| 1-year | 2539 | 492 | 3.17(2.84, 3.53) | 1098 | 234 | 2.44(2.08, 2.85) |
| 3-year | 2423 | 376 | 3.19(2.80, 3.65) | 1050 | 186 | 2.47(2.05, 2.98) |
| 5-year | 2262 | 215 | 3.31(2.74, 4.00) | 974 | 110 | 2.07(1.61, 2.68) |

Supplementary Table 8: Landmark Analysis of LPAI and All-Cause Mortality in ARIC

^1^Adjusted for Visit 5 covariates: chronological age, gender, race, education level, study center, smoking status, alcohol use, body mass index, hypertension, diabetes, eGFR, and physical activity. N represents number of participants with complete data for each outcome and all model covariates; Participants who remained alive and eligible for follow-up at each respective landmark were included in the analysis. N cases indicate the number of deaths observed during follow-up after excluding participants who died before the corresponding landmark.


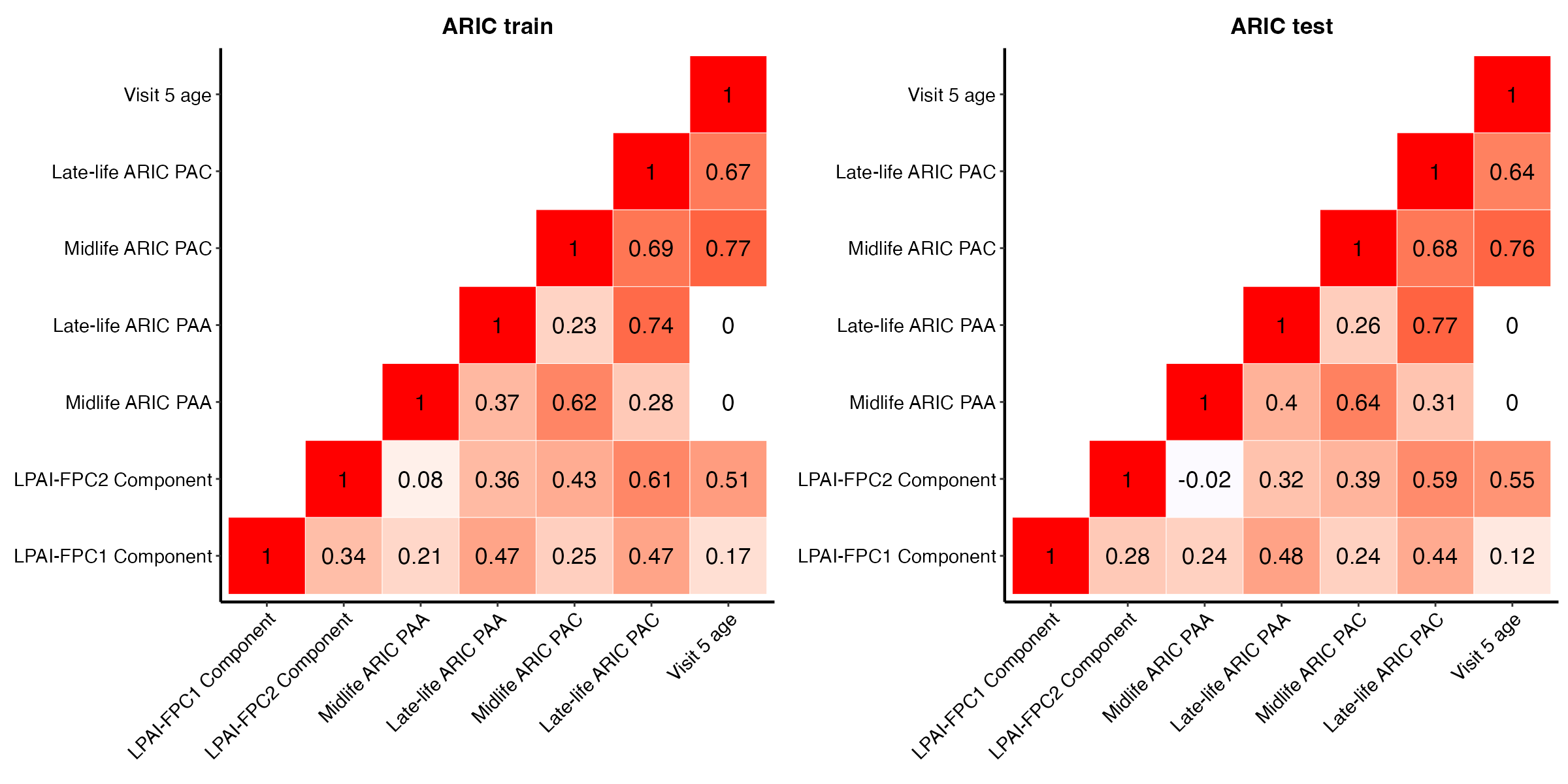


Supplementary Figure 1: Pearson correlations between the two LPAI components and related aging measures in ARIC. Each LPAI component was derived as the product of its FPC1 or FPC2 score and corresponding Cox coefficient. Correlations are shown with chronological age (Visit 5) and previously defined proteomic aging measures (PAAs and PACs).


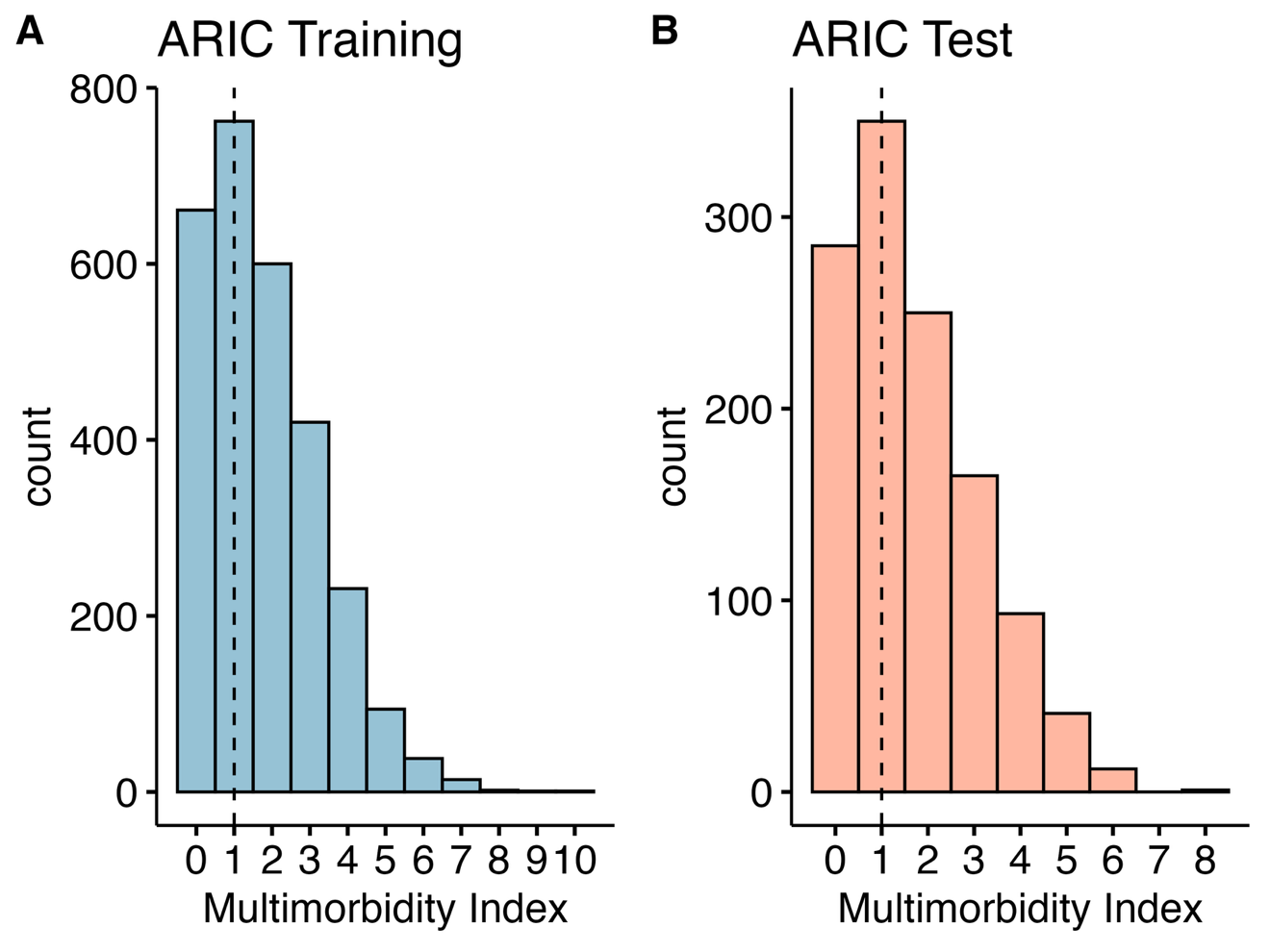


Supplementary Figure 2: Distribution of multimorbidity index in the ARIC cohort A. Histogram of the multimorbidity index in the training set Histogram of the multimorbidity index in the test set. Vertical dashed lines represent the median value in each set.


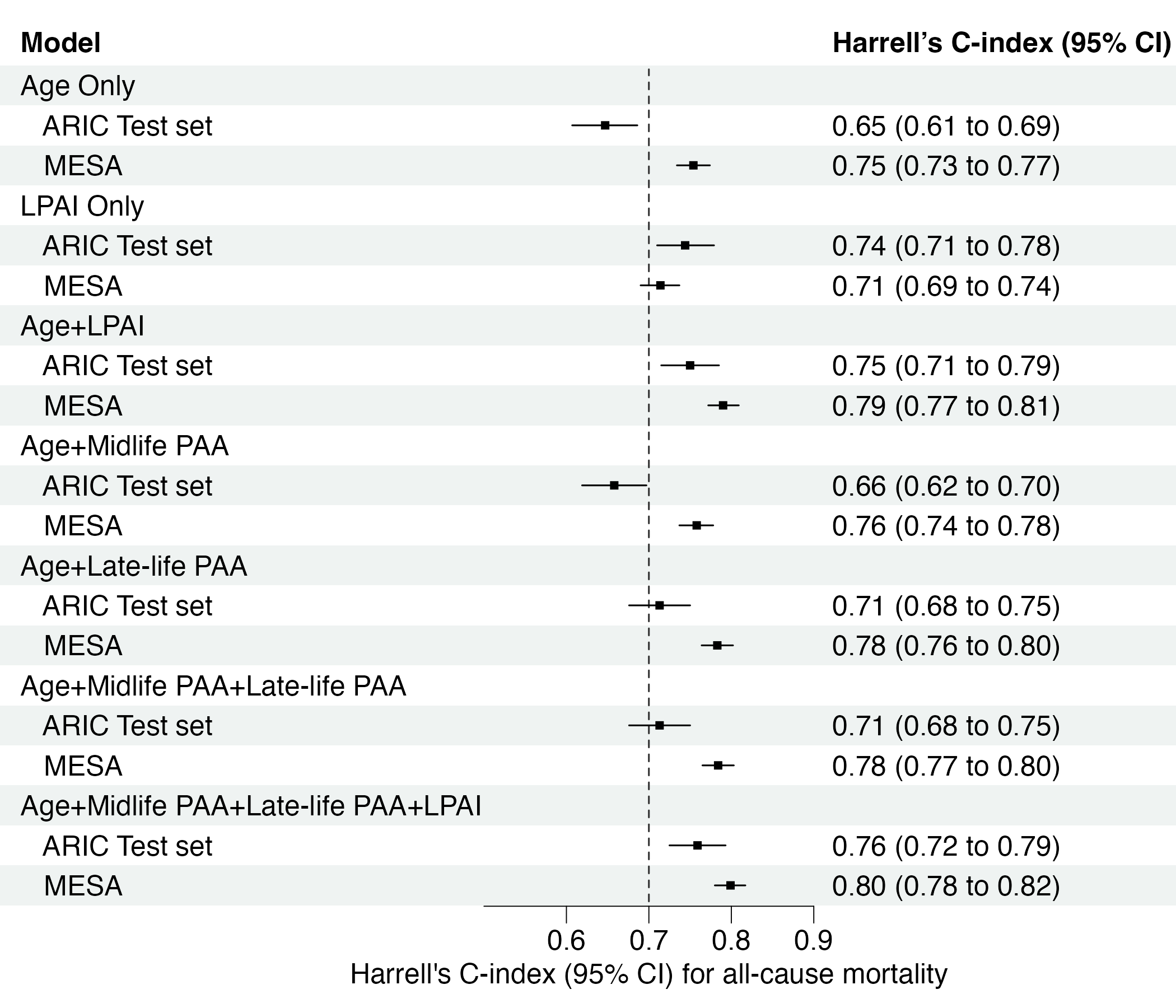


Supplementary Figure 3: Harrell’s C-indices for all-cause mortality prediction models with different combinations of aging predictors in ARIC Test set and MESA. Model in ARIC was examined among participants that were not part of the training set for either the midlife or late-life PAC.

Abbreviations: CI, confidence interval; HR, hazard ratio; PAA, Proteomic Age Acceleration; SD, standard deviation


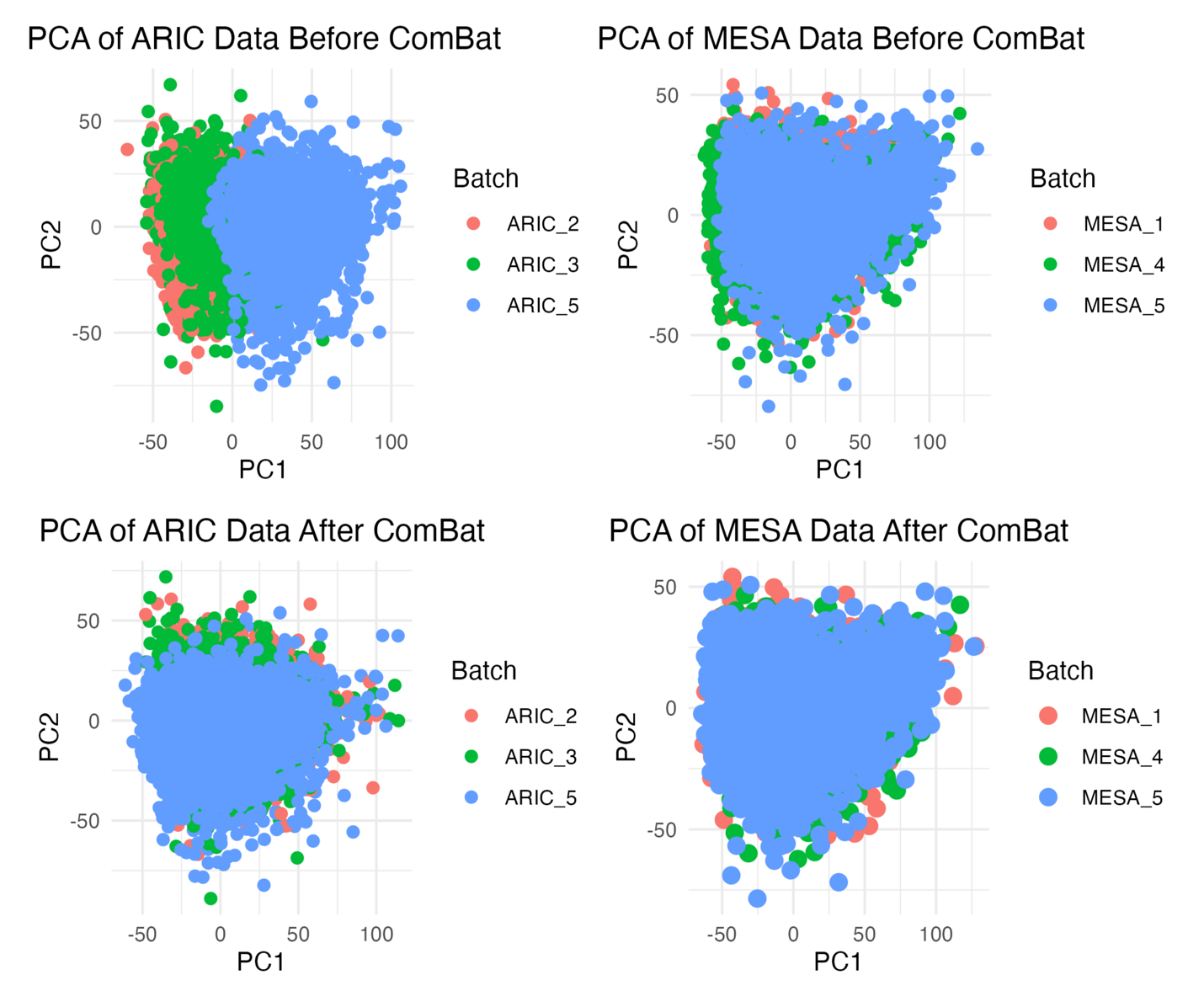


Supplementary Figure 4. PCA plots of ARIC and MESA proteomic data before and after ComBat batch correction. PCA: Principle Component Analysis


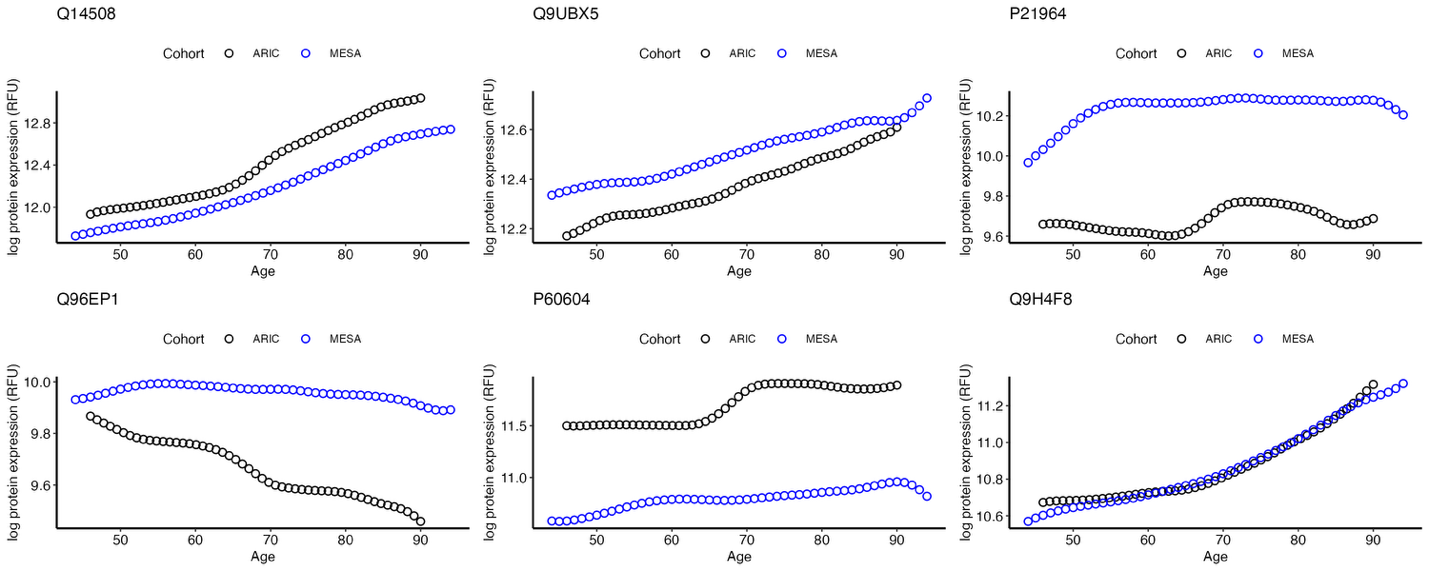


Supplementary Figure 5. Mean protein expression trajectories estimated by FPCA in the ARIC and MESA cohorts.

Mean trajectories of proteins with the three largest weights on FPC1 and FPC2 in the LPAI model, estimated using functional principal component analysis (FPCA) applied separately within each cohort, with black circles for ARIC and blue circles for MESA.


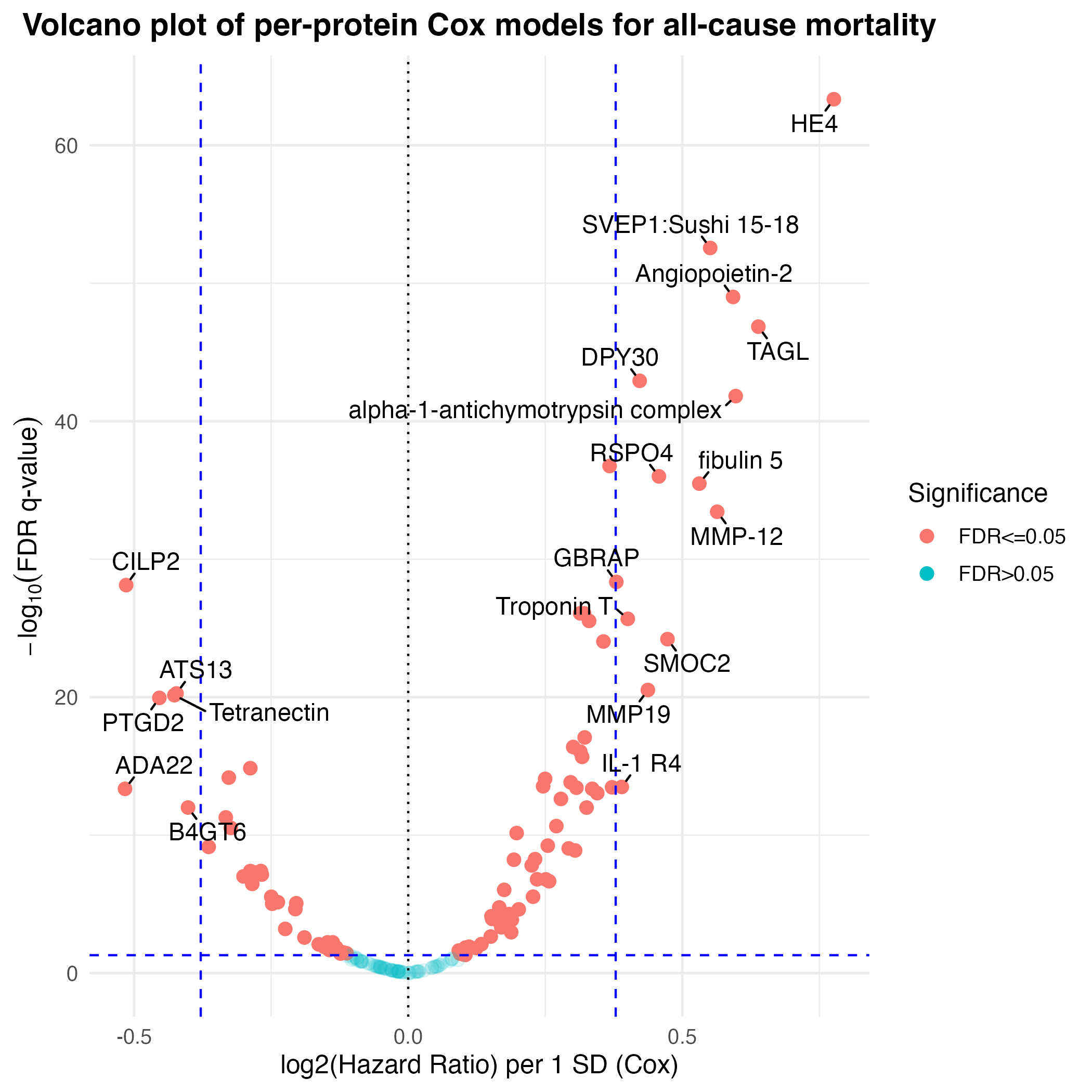


Supplementary Figure 6: Associations between LPAI proteins and all-cause mortality in ARIC.

Volcano plot showing Cox proportional hazards model results for the 181 proteins contributing to LPAI measured at Visit 5 in the ARIC cohort. Models were adjusted for age and sex. The x-axis represents the log₂(hazard ratio) per 1-SD increase in protein level, and the y-axis shows the –log₁₀(FDR q-value). Vertical dotted lines indicate HR thresholds of 1.3 and 0.77, and the horizontal dashed line marks the FDR significance threshold (q = 0.05). Proteins exceeding both criteria are labeled.
